# Planarization of Twisted Push–Pull Probes by Stretching Rather than by Compression: Core-Substituted Fluorescent Flippers as Materials Mechanosensors

**DOI:** 10.1021/jacsau.5c00579

**Published:** 2025-08-05

**Authors:** Khurnia Krisna Puji Pamungkas, Riku Yamamoto, Maxime Vonesch, Naomi Sakai, Yoshimitsu Sagara, Stefan Matile

**Affiliations:** ‡ Department of Organic Chemistry, University of Geneva, 1211 Geneva, Switzerland; ⊥ National Centre of Competence in Research (NCCR) Molecular Systems Engineering, 4002 Basel, Switzerland; § Department of Materials Science and Engineering, Institute of Science Tokyo, 2-12-1 Ookayama, Meguro-ku, Tokyo 152-8550, Japan; ¶ Research Center for Autonomous Systems Materialogy (ASMat), Institute of Science Tokyo, 4259 Nagatsuta-cho, Midori-ku, Yokohama, Kanagawa 226-8501, Japan

**Keywords:** Fluorescent probes, polymers, mechanosensitivity, mechanophores, planarizable push−pull probes, compression, stretching, torsion angles, twisted aromatics, deplanarization, chalcogen bonds

## Abstract

Fluorescent flippers are twisted push–pull mechanophores that report planarization with red-shifted absorption and an increase in fluorescence intensity and lifetime. Until today, their planarization by physical forces has focused on compression to visualize physical forces in biology. Here, we show that planarization can also be achieved by stretching of flipper probes that are equipped with tethers in their core and to visualize mechanical stress in polymeric materials. The synthesis of dithieno­[3,2-*b*:2’,3′-*d*]­thiophene dimers with alcohols extending from their twisted core is accomplished in 17 steps. Covalently integrated into polyurethanes, these core-substituted flippers exhibit an excitation-wavelength-dependent fluorescence enhancement upon polymer stretching, validating their mode of action. Noncovalently interfaced flipper controls are much less responsive. In light of the importance their fluorogenic compression has reached in biology, synthetic access to flippers that can be stretched rather than compressed and use in materials rather than life sciences opens up significant, fundamentally new perspectives for flipper research.

## Graphical abstract

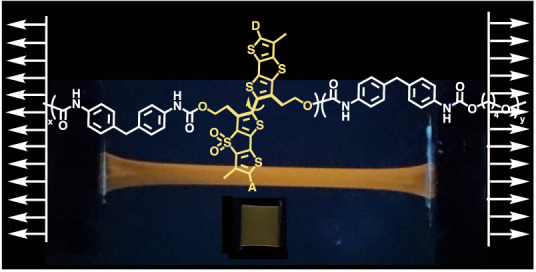

Colorimetric, fluorescence, and luminescence sensing of strain in polymeric materials presents promising applications, including plastic fatigue detection and tamper-proof materials, and has been developed through various strategies.^1–8^ The most common approaches involve mechanophores, such as spiropyranes, which change color upon covalent bond cleavage in response to mechanical stress.^9–18^ Additionally, other methods relying on weaker forces are actively explored, such as those based on aggregation,^2,19^ host–guest chemistry,^20^ rotaxanes,^8,21–24^ cyclophanes,^25–28^ bending,^29–31^ twisting,^32–35^ and so on. The twisting of mechanophores operates by changes in torsion angles that affect the conjugation of the π-system and, consequently, the optical properties. This twisting relates to one part of the more advanced mechanism of the flipper probes we developed to image physical forces in living systems.^36^

For the design of fluorescent flippers, the bioinspired^37–42^ concept of planarizable push–pull probes has been formulated.^43^ Planarization of these twisted probes **t** in equilibrium in the ground state by physical forces not only increases conjugation but also turns on a push–pull macrodipole that red shifts the absorption maximum and increases the fluorescence intensity and lifetime (Figure 1a). The specific design of fluorescent flippers is centered around two dithieno­[3,2-*b*:2’,3′-*d*]­thiophenes^44^ that are twisted out of coplanarity by repulsion between central methyl substituents and σ holes on the endocyclic central sulfur atoms (Figure 1a). Essential endocyclic acceptors and donors^45^ are complemented by exocyclic acceptors and noncovalent chalcogen-bonding^46–50^ turn-on^51^ donors.^36^ Mechanical flipper planarization brings donor and acceptors into conjugation and turns on chalcogen-bonding donors, as best appreciated in mesomer **pm**. The result is a push–pull fluorophore^52,53^ with red-shifted absorption and increased fluorescence intensity.^36,54^

**Figure 1:**
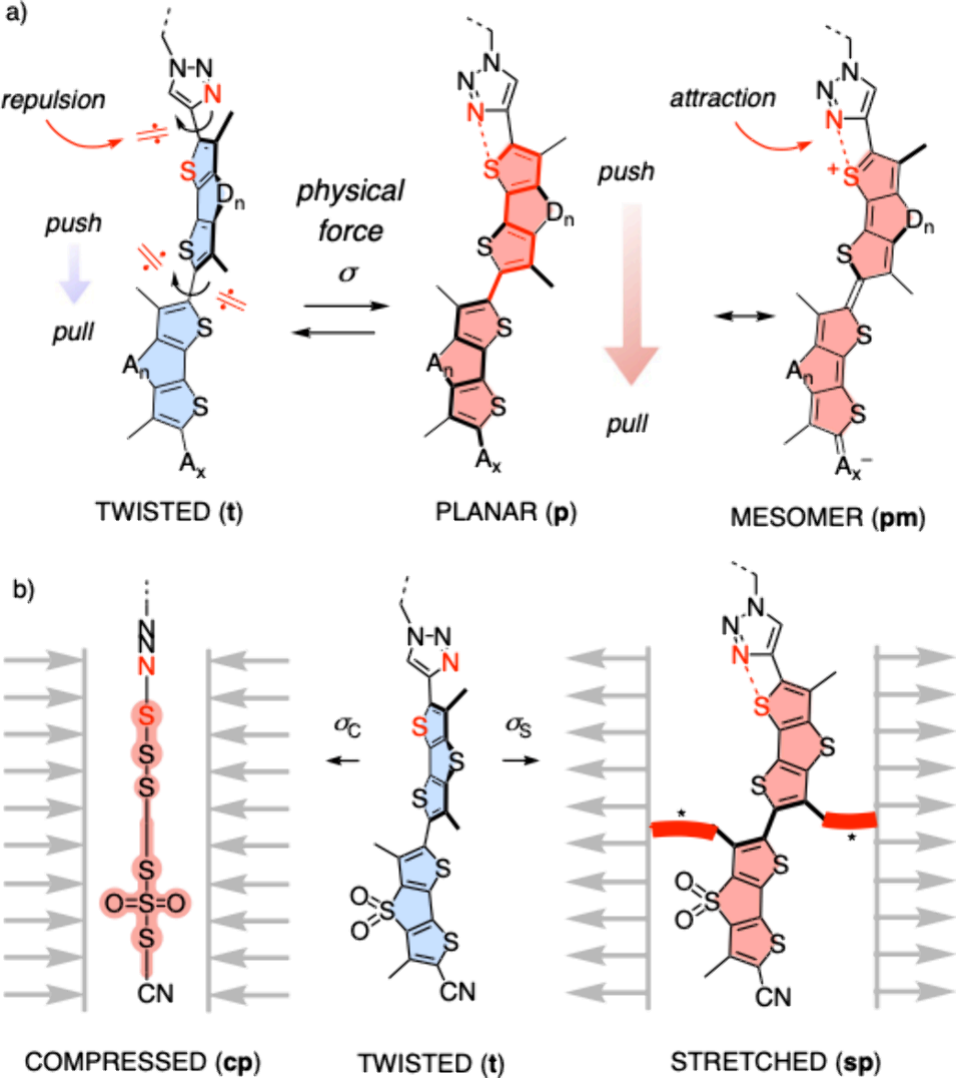
(a) Planarization of twisted push–pull probes **t** by physical forces σ affords the push–pull system **p** including mesomer **pm**. (b) Previous work focused on planarization by compression (**cp**). Here we explore planarization by stretching (**sp**), which requires substituents in the flipper core to covalently attach the stretchable substrate of interest (*).

Flipper planarization is conceivable by either compression σ_c_ to give **cp** or stretching σ_s_ to give **sp** (Figure 1b). Flipper compression has been achieved by increasing the order or tension in lipid bilayer membranes. From these results, small-molecule probes to enable tension imaging in essentially any membrane of interest within living cells have been developed and made available for the community to enable mechanobiology research worldwide.^36,55,56^ The complementary flipper planarization by stretching remains unexplored (Figure 1b). For effective planarization by stretching, pulling forces must be applied almost perpendicular to the flipper axis. This requires the tethers to be attached at the twisted flipper core rather than at the periphery (Figure 1b*). Such substitution in the flipper core, however, is synthetically demanding. The objectives of this study were to design and synthesize core-substituted flippers and investigate their mechanophoric properties after incorporation into segmented polyurethane as stretchable model substrates.

Flipper **1** was designed to enable planarization by stretching (Scheme 1). Compared with established membrane tension probes, flipper **1** preserves all essential motifs and adds two alcohols tethered to the core. This modification was not expected to negatively impact mechanosensitivity, according to our previous studies on the effects of substituting methyl groups.^57^ The triazole donor is terminated with an alkyl chain to enhance the solubility and minimize tilting in crowded environments.

**Scheme 1:**
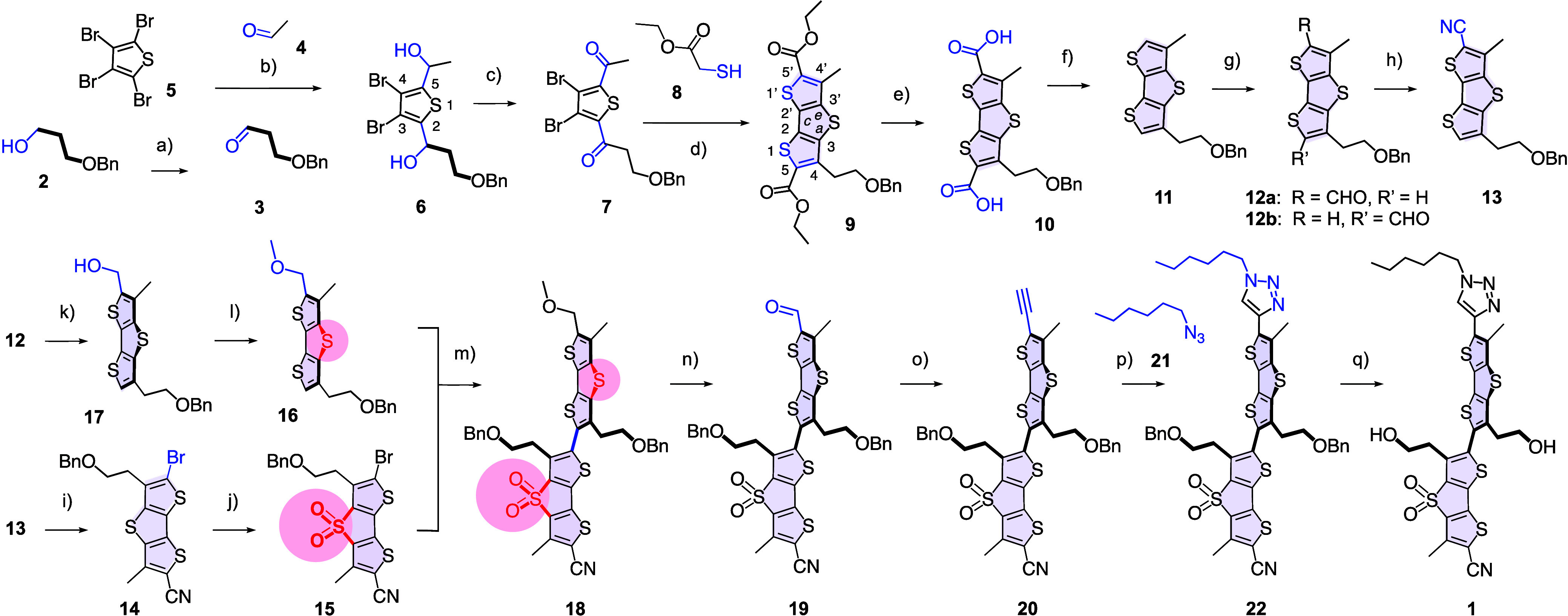
Synthesis of Core-Substituted Flippers^a^

a (a) TEMPO, diacetoxyiodobenzene, CH_2_Cl_2_, rt, 16 h, 67%. (b) 1. **4**, *n*-BuLi, 2. **5**, 3. *n*-BuLi, 4. **3**, −78 °C to rt, 18 h. (c) Dess-Martin periodinane, CH_2_Cl_2_, rt, 1 h, 56% (2 steps). (d) K_2_CO_3_, EtOH, reflux, 3 h, 78%. (e) KOH, EtOH, reflux, 18 h, 61%. (f) Ag_2_CO_3_, AcOH, DMSO, 120 °C, 18 h, 55%. (g) POCl_3_, DMF, 50 °C, 3 h, 63%, **12a**/**12b** = 3:1. (h) NaN_3_, TsOH, CH_3_CN, rt, 20 min, 53%. (i) NBS, DMF, 80 °C, 3 h, 68%. (j) mCPBA, CH_2_Cl_2_, rt, 18 h, 81%. (k) NaBH_4_, DMF, 80 °C, 1 h, 44%. (l) NaH, MeI, THF, 0 °C to rt, 18 h, 61%. (m) LDA, Bu_3_SnCl, Pd­(PPh_3_)_4_, CsF, CuI, DMF, 65 °C, 4 d, 55%. (n) DDQ, CH_2_Cl_2_/H_2_O (5:1), rt, 1 h, 94%. (o) Bestmann-Ohira reagent, K_2_CO_3_, MeOH/THF 1:2, 45 °C, 74 h, 35% (conversion yield 43%). (p) **21**, CuSO_4_·5H_2_O, NaAsc, TBTA, THF/CH_2_Cl_2_/H_2_O 3:3:1, rt, 2 h, 70%. (q) BBr_3_, CH_2_Cl_2_, −78 °C to rt, 2 h, 75%.

The synthesis of flipper **1** was achieved in 17 steps (Scheme 1). The main challenge was to construct and work with dithieno­[3,2-*b*:2’,3′-*d*]­thiophenes with different substituents at positions 4 and 4’. The new flipper core substituents were prepared from alcohol **2** and integrated at the very beginning of the synthesis. Oxidation gave aldehyde **3** with a benzyl protected terminal functional group. Stepwise Li/Br exchange allowed to add first acetaldehyde **4** and then aldehyde **3** in positions 2 and 5 of tetrabromothiophene **5**. Dess-Martin oxidation of the resulting diol **6** afforded unsymmetrical diketone **7** in respectable 56% yield over 2 steps.

An intriguing cascade reaction covering electrophilic aromatic substitution, enolate addition, and dehydration merged diketone **7** and thiol **8** into the dithieno­[3,2-*b*:2’,3′-*d*]­thiophene core **9** with two different substituents in positions 4 and 4’. Ester hydrolysis followed by decarboxylation of **10** liberated the positions 5 and 5′ in the first key intermediate **11**. Vilsmeier–Haack reaction generated a regioisomeric mixture **12** in 63% yield with a formyl group on the methyl side being the major isomer (3:1).

With aldehyde **12**, the synthesis of the dithieno­[3,2-*b*:2’,3′-*d*]­thiophene donor and the dithieno­[3,2-*b*:2’,3′-*d*]­thiophene-*S,S*-dioxide acceptor diverged. The synthesis of the dithieno­[3,2-*b*:2’,3′-*d*]­thiophene donor required first the demanding conversion of the 5′-aldehyde **12** with sodium azide to access regioisomer-free nitrile **13** in 53% yield. Subsequent bromination of 5′-nitrile **13** at position 5 proceeded smoothly with 68% yield, and oxidation of the pseudosulfide bridge in **14** with mCPBA could be realized in 81% yield.

The resulting bright dithieno­[3,2-*b*:2’,3′-*d*]­thiophene-*S,S*-dioxide acceptor **15** was ready for Stille coupling with the dithieno­[3,2-*b*:2’,3′-*d*]­thiophene donor **16**. This donor **16** was obtained from aldehyde **12** in two steps through reduction with sodium borohydride, giving the regioisomer-free primary alcohol **17**, followed by methylation with NaH and MeI.

Stille coupling of acceptor **15** and donor **16** gave twisted flipper scaffold **18** in up to 55% yield. Oxidative conversion of methyl ether **18** into aldehyde **19** with DDQ was unproblematic. The terminal alkyne **20** was introduced using the Bestmann-Ohira reagent, that is dimethyl­(1-diazo-2-oxopropyl)­phosphonate. CuAAC with alkylazide **21** gave the protected final product **22** in 70% yield. Benzyl deprotection with BBr_3_ liberated the tethered primary alcohols in the twisted core of final flipper **1**.

To prevent aggregation-induced quenching^58,59^ and minimize the disturbance of polymer properties, flipper concentration should ideally be kept low. Thus, flipper **1** with a diol in the core was inserted in the segmented polyurethane as a trace component by reacting with diisocyanate **23** besides poly-THF **24** (*M*_n_ = 2000) and butanediol **25** (Figure 2a). Polymerization proceeded in THF at room temperature (rt) first for 3 h in the absence and then for 24 h in the presence of **25**. The resulting polyurethanes **26** had an *M*_n_ = 67000 with *x*/*y*/*z* = 1.0:2.2:0.002 (6.58 × 10^–7^ mol/g, 0.047 wt % flipper). Since the amount of added flipper **1** is tiny, the corresponding ^1^H NMR peaks cannot be observed on the ^1^H NMR chart (Figure S1). However, comparison between the UV–vis absorption spectra of flipper **1** and polyurethane **26** clarified incorporation of the flipper into the polymer chain (Figure S2). The thermogravimetric analysis (TGA) and differential scanning calorimetry (DSC) revealed that the thermal properties of polyurethane **26** are similar to those of previously reported segmented polyurethane.^21,22,25,26^ A clear decrease in weight starts from ca. 240 °C on the TGA trace (Figure S3). An exothermic peak was observed around 100 °C on the DSC curve on the first cooling and an endothermic peak was found around 170 °C (Figure S4). These peaks are ascribed to crystallization and melting of the hard segment of polyurethane **26**, respectively. Films having a thickness of ∼ 85 μm were prepared by spreading the polymer solution and evaporating the solvent. They were cut into dog-bone shapes. Tensile tests reveal an elongation at break of ∼ 651%, a stress at break close to 54.4 MPa, and a Young’s modulus of ca. 10.1 MPa (Figure S5 and Table S1).

**Figure 2:**
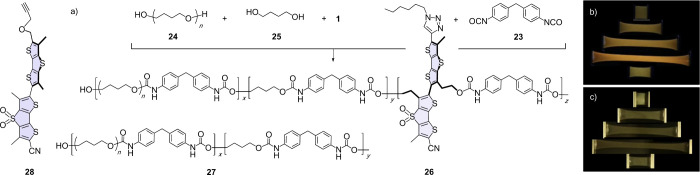
(a) Covalent integration of core-substituted flippers **1** into polyurethanes **26** (*M*_n_ = 67000 with *x*/*y*/*z* = 1.0:2.2:0.002), and polyurethane **27** (*M*_n_ = 51000 with *x*/*y* = 1.0:2.0) used for the noncovalent integration of flippers **1**, **22** or **28**. (b) Photos of polyurethanes **26** before (top), after (bottom) and during stretching by 200%, 400% and 600%, excited at 365 nm, and (c) same without excitation.

To compare covalent stretching with noncovalent interfacing of twisted flippers, thin films were also prepared from a mixture of unlabeled polyurethanes **27** (*M*_n_ = 51000, *x*/*y* = 1.0:2.0) and flipper **28**,^59^ **22** or **1** (Figure 2a). The concentrations of flippers in the films were kept in the same range, i.e., 6.58 × 10^–7^ mol/g, corresponding to 0.038, 0.059, and 0.047 wt %, respectively.

Because of their significance to image forces in biology, the photophysical response to flipper planarization has been studied in detail.^36^ In the relaxed twisted form, the flipper probe absorbs at relatively high energy with a maximum of just above 400 nm (Figure 3, blue). The equally twisted Franck–Condon (FC) state planarizes within a few picoseconds into a planar intramolecular charge transfer (PICT) state, characteristic of push–pull chromophores. Emission from the PICT state is thus strongly red-shifted, maximal around 600 nm, compared to excitation just above 400 nm. Ultrafast excited-state planarization is in competition with radiation-free vibrational relaxation, which lowers fluorescence intensity, lifetime and quantum yield.

**Figure 3:**
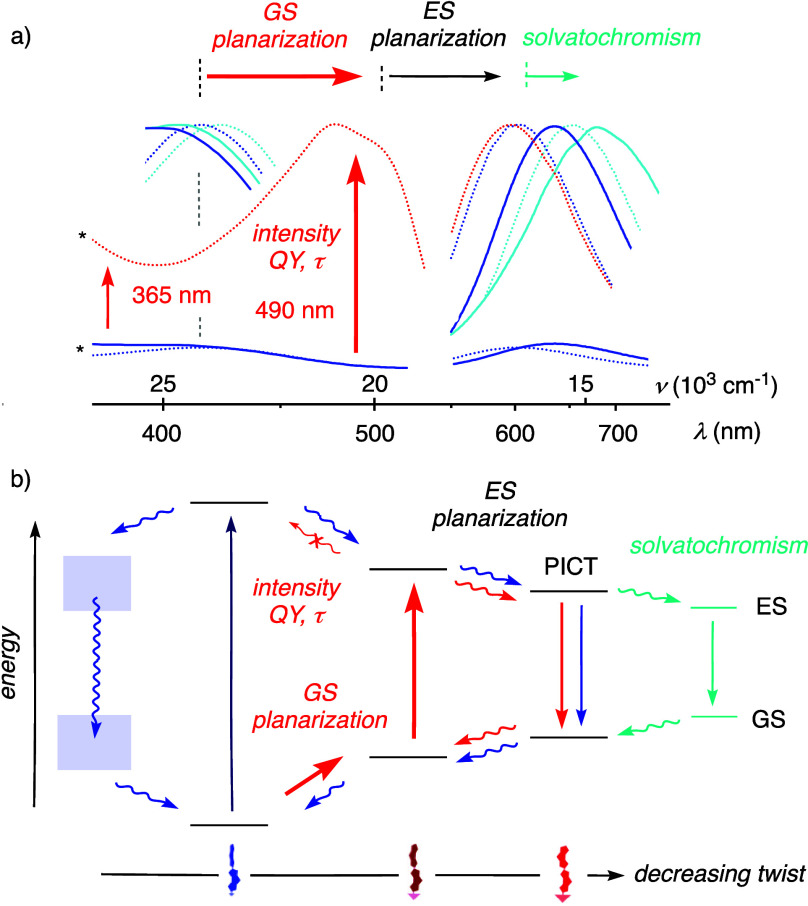
Excitation and emission spectra (a) and energy diagram (b) of twisted flippers in solution (THF blue, DMSO cyan) and planarized flippers in ordered lipid bilayer membranes (red), with indication of the origin of the wavelength dependence of intensity changes upon planarization (red vertical arrows) and the shifts of excitation (red) and emission maxima (black, cyan horizontal arrows). (a) Normalized excitation and emission spectra of **1** (solid) compared to original flippers (dotted) in THF (blue; *estimated relative intensities compared to original flippers in ordered membranes), DMSO (cyan), and original flippers in ordered membranes (red). (b) Putative energy diagram of flipper probes in the ground (GS) and excited states (ES) as a function of decreasing twist.

Unlike most other environment-sensitive fluorophores, flipper probes sense the physical forces in the ground state (GS) and not in the excited state (ES).^36^ Mechanical flipper planarization in equilibrium in the GS first raises the HOMO and lowers the LUMO, which accounts for the red-shifted excitation maximum (Figure 3, red). The following ES planarization and emission from the PICT state is like that for twisted flippers. The emission wavelength is not mechanosensitive for this reason. Positive solvatochromism of the emission maximum is the same as for all PICT states. It reports simultaneously on the polarity of the environment, unrelated to mechanosensing.^60^ The strong increase in fluorescence intensity and lifetime in response to GS planarization occurs because the equally planarized FC state disfavors nonemissive processes in favor of ES planarization into the emissive PICT state (Figure 3, red). Because of the shift of the excitation maximum, this fluorescence enhancement is wavelength dependent. Wavelength-dependent intensity increases can thus provide experimental evidence for flipper planarization in the ground state, independent of parameters that can be difficult to control in complex systems such as effective concentrations or polarity of the environment.

Excitation and emission maxima of twisted core-substituted flippers **1** in solution (Figure 3a, blue, solid) did not differ much from those of original flipper derivatives **28** (Figure 3a, blue, dotted). While core substitution might contribute,^57^ the small differences mostly originated from the different exocyclic donors used.^61^ More importantly, the overall preserved giant Stokes shift produced by ES planarization implied that core substitution would not interfere with mechanical GS planarization either, i.e., that flipper **1** is operational as a mechanosensor. Spectra of planarized flippers **1** in ordered lipid bilayer membranes were not recorded because they were designed to work differently, but their fluorescence spectra should resemble those of original flippers planarized in ordered membranes (Figure 3a, red).

When the polyurethane elastomer films were irradiated at 365 nm, the increase in fluorescence emission upon stretching was visible with the naked eye (Figure 2b), while absorbance decreased due to film thinning (Figure 2c). Relaxation brought the fluorescence back to low intensity. The mechanoresponsive luminescence was further investigated with *in situ* photoluminescence spectroscopy. With increasing strain, the emission intensity gradually increased upon stretching (Figure 4a, top). The rapid decrease in emission intensity was observed upon relaxation due to the stress relaxation of polyurethane elastomers (Figure 4a, bottom). Repeated stretching resulted in an initial decrease in the fluorescence intensity of the deformed film (Figure 4c). After this initial drop in responsiveness to about half, stretch relaxation cycles could be repeated more than 30 times without further loss of sensitivity (Figure 4c). The measured intensities correlated well with the applied stress (Figures 4e,f and S9).

**Figure 4:**
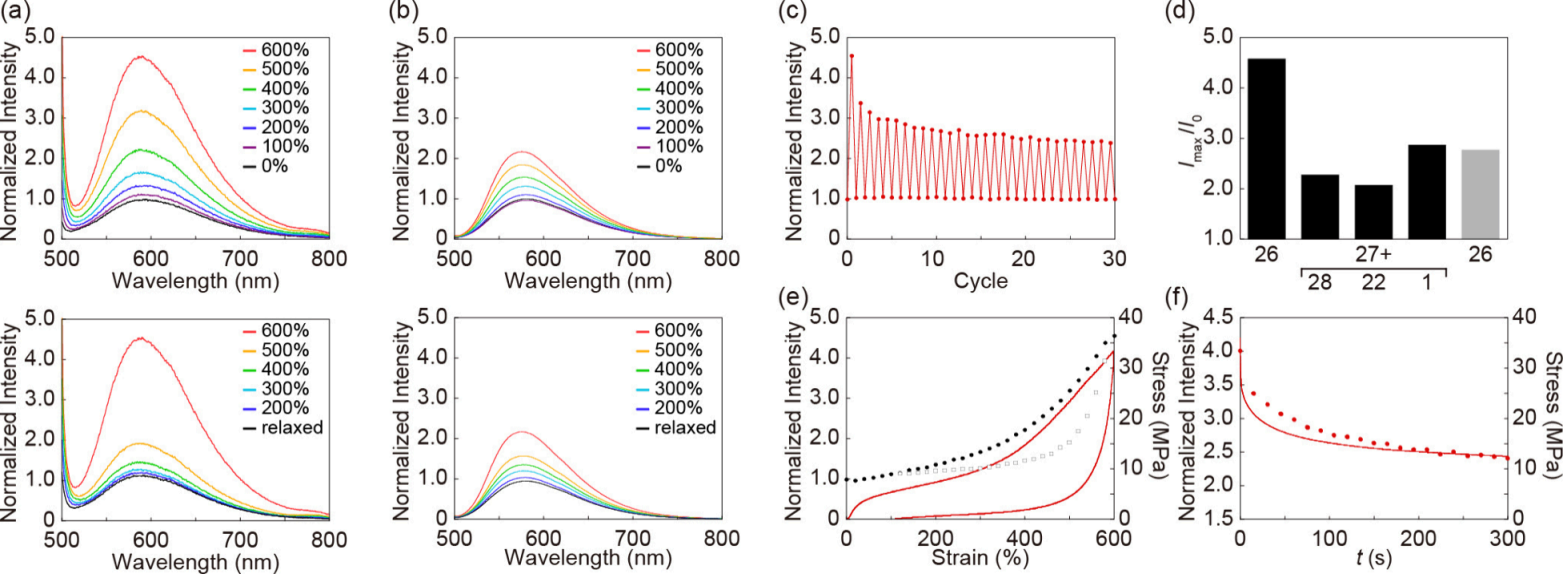
(a) Emission spectra of polyurethane **26** with covalently interfaced flippers upon stretching (top) and relaxing (bottom) the film to the indicated strains with excitation light of 490 nm. (b) Same for polyurethane **27** with noncovalently bonded flipper **28**. (c) Stretch-relax cycles for **26**. (d) Fluorescence intensity of polyurethanes at maximal (*I*_max_, 600%) and minimal stress (*I*_0_) for covalent (**26**) and noncovalent (**1**, **22**, **28**) flipper interfacing, excited at 490 nm (black) or 365 nm (gray). (e) Overlays of the stress–strain curves (lines) and the corresponding emission intensities (circles and squares). (f) Nominal stress (line) and emission intensity (circles) measured as the film is stretched to 600% strain and then held with the same strain. The emission intensities (c, e, f) were recorded at 600 nm with excitation at 490 nm.

Stretching of **26** monitored by excitation at 490 nm instead of 365 nm gave a much stronger fluorescence response (Figure 4d, gray vs black) at otherwise unchanged properties (Figures 4a, S6). Wavelength-dependent fluorescence increases upon GS planarization are a hallmark of flipper photophysics.^36^ They are the consequence of the red shift of the excitation maxima upon GS planarization of the twisted push–pull system (Figure 3). The observed wavelength dependence thus evinced that the fluorescence enhancement caused by polymer stretching originates indeed from mechanical GS planarization of the flipper by stretching (Figure 4d, gray vs black).

Stretching of **27** doped with the original flipper **28** caused a much smaller increase in fluorescence compared to the covalently interfaced flippers in **26** excited at the same 490 nm (Figure 4a, b, and d, black bars). Noncovalent interfacing of polyurethanes **27** with the synthetic intermediate **22** and the final core-substituted flipper **1** gave a similarly weak fluorescent response to stretching, one stronger and one weaker than the original **28** (Figures 4d, S7, and S8). These results supported that (i) also noncovalently interfaced flippers can be planarized, but (ii) planarization of covalently interfaced core-substituted flippers by stretching is much more effective in polymers (Figure 1b). Considering their role as negative controls with inferior performance, further discussion of the nature of planarization of noncovalently interfaced flippers is less important. Noncovalent stretching enabled by hydrogen bonds between the alcohols in the flipper core and polymer chains would be conceivable for the most responsive **1** in polyurethanes **27**, as at least two hydrogen bonds in apolar polymers should be stronger than the <20 kJ mol^–1^ needed to planarize flippers.^57^ Partial planarization by compression along mechanically aligned polymer chains was also conceivable,^62–65^ and different local environments within the polymers could also contribute to the probes’ response.^66^

Overall, this study reports synthetic access to core-substituted flippers. This is of interest to covalently attach polymer chains, for instance, on both sides of the twisted core of the mechanophore. Flipper planarization by stretching then visualizes the stress applied to the polymer as a wavelength-dependent increase in the fluorescence intensity. Compared to other mechanophores that operate by monomer bending^29–31^ and twisting,^32–35^ flipper probes are unique because they operate by a different, bioinspired mode of action that combines mechanical planarization in equilibrium in the ground state with a push–pull switch (Figure 1). This produces a large red shift of the excitation maximum in the visible range, with increasing rather than vanishing^29,30^ intensity, compatible with excitation rather than emission wavelength-dependent detection. Ground-state mechanosensitivity reported in the excitation spectrum leaves the emission window free for the simultaneous but independent detection of the polarity of the environment by positive solvatochromism (Figure 3).^60,67^ While mechanophores that operate by bending without push–pull switch failed to planarize in the ground state in biological membranes,^68^ the unique properties obtained with flipper probes created a significant demand^36,69–71^ that made their commercialization worthwhile, despite laborious syntheses involving ≥15 steps. This study now opens up fundamentally new directions for flipper research, stretching not pressing, applied to materials, not biology. We hope that the concerned communities will find the availability of distinct flipper chemistry equally inspiring.

## Supplementary Material



## Data Availability

The data that support the findings of this study are openly available in Zenodo at 10.5281/zenodo.15624958.

## Notes

Detailed experimental procedures, materials and methods, procedures for multistep synthesis of core-substituted flippers, characterization of all new compounds, polymer synthesis with covalent and noncovalent mechanophore integration, preparation of polymer films, thermal polymer properties, mechanical polymer properties, mechanoresponsive polymer fluorescence (PDF)

## +

Universitas Gadjah Mada, Sleman, Yogyakarta 55281, Indonesia

## †

Chemspeed Technologies AG, Wölferstrasse 8, 4414 Füllinsdorf, Switzerland

## References

[1] Chen Y., Mellot G., van Luijk D., Creton C., Sijbesma R. P. (2021). Mechanochemical
Tools for Polymer Materials. Chem. Soc. Rev..

[2] Schrettl S., Lattuada M., Fromm K. M., Simon Y. C., Di Giannantonio M., Verde-Sesto E., Sagara Y., Neumann L. N., Lavrenova A., Karman M., Calvino C., Balkenende D. W.R., Weder C. (2019). Functional Polymers Through Mechanochemistry. Chimia.

[3] Li J., Nagamani C., Moore J. S. (2015). Polymer Mechanochemistry: From Destructive
to Productive. Acc. Chem. Res..

[4] Black A. L., Lenhardt J. M., Craig S. L. (2011). From Molecular
Mechanochemistry to
Stress-Responsive Materials. J. Mater. Chem..

[5] Lin Y., Kouznetsova T. B., Foret A. G., Craig S. L. (2024). Solvent Polarity
Effects on the Mechanochemistry of Spiropyran Ring Opening. J. Am. Chem. Soc..

[6] Traeger H., Kiebala D., Calvino C., Sagara Y., Schrettl S., Weder C., Clough J. M. (2023). Microscopic Strain Mapping in Polymers
Equipped with Non-Covalent Mechanochromic Motifs. Mater. Horiz..

[7] Traeger H., Sagara Y., Kiebala D. J., Schrettl S., Weder C. (2021). Folded Perylene
Diimide Loops as Mechanoresponsive Motifs. Angew.
Chem. Int. Ed..

[8] Muramatsu T., Okado Y., Traeger H., Schrettl S., Tamaoki N., Weder C., Sagara Y. (2021). Rotaxane-Based Dual Function Mechanophores
Exhibiting Reversible and Irreversible Responses. J. Am. Chem. Soc..

[9] Overholts A. C., Granados Razo W., Robb M. J. (2023). Mechanically Gated Formation of Donor-Acceptor
Stenhouse Adducts Enabling Mechanochemical Multicolour Soft Lithography. Nat. Chem..

[10] Watabe T., Otsuka H. (2023). Swelling-Induced Mechanochromism
in Multinetwork Polymers. Angew. Chem. Int.
Ed..

[11] Zhang C., Kouznetsova T. B., Zhu B., Sweeney L., Lancer M., Gitsov I., Craig S. L., Hu X. (2025). Advancing the Mechanosensitivity
of Atropisomeric Diarylethene Mechanophores through a Lever-Arm Effect. J. Am. Chem. Soc..

[12] Sun Z.-Y., Li Y., Wu M., He W., Yuan Y., Cao Y., Chen Y. (2024). A Rhodamine-Spiropyran Conjugate Empowering Tunable Mechanochromism
in Polymers under Multiple Stimuli. Angew. Chem.
Int. Ed..

[13] Sun Y., Wang K., Huang X., Wei S., Contreras E., Jain P. K., Campos L. M., Kulik H. J., Moore J. S. (2024). Caged AIEgens:
Multicolor and White Emission Triggered by Mechanical Activation. J. Am. Chem. Soc..

[14] Centellas P. J., Mehringer K. D., Bowman A. L., Evans K. M., Vagholkar P., Thornell T. L., Huang L., Morgan S. E., Soles C. L., Simon Y. C., Chan E. P. (2024). Mechanochemically Responsive Polymer
Enables Shockwave Visualization. Nat. Commun..

[15] Xu D., Liu W., Tian S., Qian H. (2025). Versatile Mechanochemical Reactions
Via Tailored Force Transmission in Mechanophores. Angew. Chem. Int. Ed..

[16] Gridneva T., Khusnutdinova J. R. (2025). Functional Coordination Compounds for Mechanoresponsive
Polymers. Chem. Commun..

[17] Osler S. K., Ballinger N. A., Robb M. J. (2025). The Role of Torsion on the Force-Coupled
Reactivity of a Fluorenyl Naphthopyran Mechanophore. J. Am. Chem. Soc..

[18] Steponavičiu̅tė M., Majee D., Zhao B., Ungur L., Presolski S. (2025). Expanding
the Molecular Switches Toolbox: Photoreloadable Dithienylethene Mechanophores. Angew. Chem. Int. Ed.

[19] Han T., Liu L., Wang D., Yang J., Tang B. Z. (2021). Mechanochromic Fluorescent
Polymers Enabled by AIE Processes. Macromol.
Rapid Commun..

[20] Das A. D., Mannoni G., Früh A. E., Orsi D., Pinalli R., Dalcanale E. (2019). Damage-Reporting
Carbon Fiber Epoxy Composites. ACS Appl. Polym.
Mater..

[21] Sagara Y., Karman M., Verde-Sesto E., Matsuo K., Kim Y., Tamaoki N., Weder C. (2018). Rotaxanes as Mechanochromic Fluorescent
Force Transducers in Polymers. J. Am. Chem.
Soc..

[22] Sagara Y., Karman M., Seki A., Pannipara M., Tamaoki N., Weder C. (2019). Rotaxane-Based Mechanophores Enable
Polymers with Mechanically Switchable White Photoluminescence. ACS Cent. Sci..

[23] Hiratsuka K., Muramatsu T., Seki T., Weder C., Watanabe G., Sagara Y. (2023). Tuning the Mechanoresponsive Luminescence
of Rotaxane
Mechanophores by Varying the Stopper Size. J.
Mater. Chem. C.

[24] Mori R., Weder C., Sagara Y. (2023). Mechanical (De)­Activation of Rotaxane
Mechanophores: Axle Length Matters. Macromolecules.

[25] Sagara Y., Traeger H., Li J., Okado Y., Schrettl S., Tamaoki N., Weder C. (2021). Mechanically
Responsive Luminescent
Polymers Based on Supramolecular Cyclophane Mechanophores. J. Am. Chem. Soc..

[26] Thazhathethil S., Muramatsu T., Tamaoki N., Weder C., Sagara Y. (2022). Excited State
Charge-Transfer Complexes Enable Fluorescence Color Changes in a Supramolecular
Cyclophane Mechanophore. Angew. Chem. Int. Ed..

[27] Shimizu S., Yoshida H., Mayumi K., Ajiro H., Sagara Y. (2023). Mechanochromic
Luminescence of Phase-Separated Hydrogels That Contain Cyclophane
Mechanophores. Mater. Chem. Front..

[28] Thazhathethil S., Thuluvanchery F. S., Shimizu S., Scarlat I., Clough J. M., Weder C., Sagara Y. (2024). Ring-Size Dependent Ratiometric Photoluminescence
of Cyclophane Mechanophores. J. Mater. Chem.
C.

[29] Suga K., Yamakado T., Saito S. (2023). Dual Ratiometric
Fluorescence Monitoring
of Mechanical Polymer Chain Stretching and Subsequent Strain-Induced
Crystallization. J. Am. Chem. Soc..

[30] Kotani R., Yokoyama S., Nobusue S., Yamaguchi S., Osuka A., Yabu H., Saito S. (2022). Bridging Pico-to-Nanonewtons
with a Ratiometric Force Probe for Monitoring Nanoscale Polymer Physics
before Damage. Nat. Commun..

[31] Ofodum N. M., Qi Q., Chandradat R., Warfle T., Lu X. (2024). Advancing Dynamic Polymer
Mechanochemistry through Synergetic Conformational Gearing. J. Am. Chem. Soc..

[32] Doan H., Raut S. L., Yale D., Balaz M., Dzyuba S. V., Gryczynski Z. (2016). Mechanothermally Induced Conformational Switch of a
Porphyrin Dimer in a Polymer Film. Chem. Commun..

[33] Hu H., Cheng X., Wu Y., Chen M., Ma Z., Yang Q., Sijbesma R. P., Ma Z. (2024). Spirolactam and ESIPT
in a Double-Network Elastomer: Multicolor Mechanochromism and Dual-Ratiometric
Strain Sensing in Low and High Tensile Stress. Macromolecules.

[34] Hertel R., Maftuhin W., Walter M., Sommer M. (2022). Conformer
Ring Flip
Enhances Mechanochromic Performance of Ansa-Donor-Acceptor-Donor Mechanochromic
Torsional Springs. J. Am. Chem. Soc..

[35] Raisch M., Maftuhin W., Walter M., Sommer M. (2021). A Mechanochromic Donor-Acceptor
Torsional Spring. Nat. Commun..

[36] Chen X.-X., Bayard F., Gonzalez-Sanchis N., Puji Pamungkas K. K., Sakai N., Matile S. (2023). Fluorescent Flippers:
Small-Molecule
Probes to Image Membrane Tension in Living Systems. Angew. Chem. Int. Ed..

[37] Begum S., Cianci M., Durbeej B., Falklöf O., Hädener A., Helliwell J. R., Helliwell M., Regan A. C., Watt C. I. F. (2015). On the Origin and Variation of Colors
in Lobster Carapace. Phys. Chem. Chem. Phys..

[38] Gamiz-Hernandez A. P., Angelova I. N., Send R., Sundholm D., Kaila V. R. I. (2015). Protein-Induced
Color Shift of Carotenoids in β-Crustacyanin. Angew. Chem. Int. Ed..

[39] Sheves M., Nakanishi K., Honig B. (1979). Through-Space Electrostatic Effects
in Electronic Spectra. Experimental Evidence for the External Point-Charge
Model of Visual Pigments. J. Am. Chem. Soc..

[40] Kiser P. D., Golczak M., Palczewski K. (2014). Chemistry
of the Retinoid (Visual)
Cycle. Chem. Rev..

[41] Baumeister B., Matile S. (2000). Rigid-Rod β-Barrels
as Lipocalin Models: Probing
Confined Space by Carotenoid Encapsulation. Chem.—Eur. J..

[42] Winum J.-Y., Matile S. (1999). Rigid Push-Pull Oligo­(p-Phenylene)
Rods: Depolarization
of Bilayer Membranes with Negative Membrane Potential. J. Am. Chem. Soc..

[43] Fin A., Vargas Jentzsch A., Sakai N., Matile S. (2012). Oligothiophene Amphiphiles
as Planarizable and Polarizable Fluorescent Membrane Probes. Angew. Chem. Int. Ed..

[44] Strakova K., Assies L., Goujon A., Piazzolla F., Humeniuk H. V., Matile S. (2019). Dithienothiophenes at Work: Access
to Mechanosensitive Fluorescent Probes, Chalcogen-Bonding Catalysis,
and Beyond. Chem. Rev..

[45] García-Calvo J., López-Andarias J., Sakai N., Matile S. (2021). The Primary
Dipole of Flipper Probes. Chem. Commun..

[46] Biot N., Bonifazi D. (2020). Chalcogen-Bond Driven
Molecular Recognition at Work. Coord. Chem.
Rev..

[47] Scilabra P., Terraneo G., Resnati G. (2019). The Chalcogen
Bond in Crystalline
Solids: A World Parallel to Halogen Bond. Acc.
Chem. Res..

[48] Bauzá A., Mooibroek T. J., Frontera A. (2015). The Bright Future of Unconventional
σ/π-Hole Interactions. ChemPhysChem.

[49] Jovanovic D., Mohanan M. P., Huber S. M. (2024). Halogen,
Chalcogen, Pnictogen, and
Tetrel Bonding in Non-Covalent Organocatalysis: An Update. Angew. Chem. Int. Ed..

[50] Singh A., Torres-Huerta A., Meyer F., Valkenier H. (2024). Anion Transporters
Based on Halogen, Chalcogen, and Pnictogen Bonds: Towards Biological
Applications. Chem. Sci..

[51] Scheller Z. N., Mehrparvar S., Haberhauer G. (2025). Light-Induced Increase in Bond Strength—from
Chalcogen Bond to Three-Electron σ Bond upon Excitation. J. Am. Chem. Soc..

[52] Klymchenko A. S. (2017). Solvatochromic
and Fluorogenic Dyes as Environment-Sensitive Probes: Design and Biological
Applications. Acc. Chem. Res..

[53] Liu P., Miller E. W. (2020). Electrophysiology,
Unplugged: Imaging Membrane Potential
with Fluorescent Indicators. Acc. Chem. Res..

[54] Paez-Perez M., Kuimova M. (2024). Molecular Rotors: Fluorescent
Sensors for Microviscosity
and Conformation of Biomolecules. Angew. Chem.
Int. Ed..

[55] Pamungkas K. K. P., Fureraj I., Assies L., Sakai N., Mercier V., Chen X.-X., Vauthey E., Matile S. (2024). Core-Alkynylated Fluorescent
Flippers: Altered Ultrafast Photophysics to Track Thick Membranes. Angew. Chem. Int. Ed..

[56] Saidjalolov S., Chen X.-X., Moreno J., Cognet M., Wong-Dilworth L., Bottanelli F., Sakai N., Matile S. (2024). Asparagusic Golgi Trackers. JACS Au.

[57] Strakova K., Poblador-Bahamonde A. I., Sakai N., Matile S. (2019). Fluorescent
Flipper Probes: Comprehensive Twist Coverage. Chem. - Eur. J..

[58] Macchione M., Chuard N., Sakai N., Matile S. (2017). Planarizable Push-Pull
Probes: Overtwisted Flipper Mechanophores. ChemPlusChem..

[59] Bayard F., Matile S. (2024). Fluorescent Membrane
Probes Obey the Israelachvili
Rules. Helv. Chim. Acta.

[60] Sakai N., Assies L., Matile S. (2022). G-Quartets,
4-Way Junctions and Triple
Helices but Not DNA Duplexes: Planarization of Twisted Push-Pull Flipper
Probes by Surface Recognition Rather Than Physical Compression. Helv. Chim. Acta.

[61] Macchione M., Goujon A., Strakova K., Humeniuk H. V., Licari G., Tajkhorshid E., Sakai N., Matile S. (2019). A Chalcogen-Bonding
Cascade Switch for Planarizable Push-Pull Probes. Angew. Chem. Int. Ed..

[62] de
Gennes P. G. (1980). Conformations of Polymers Attached to an Interface. Macromolecules.

[63] Mackley M. (2010). Stretching
Polymer Chains. Rheol. Acta.

[64] Burba C. M., Woods L., Millar S. Y., Pallie J. (2011). Polymer Chain Organization
in Tensile-Stretched Poly­(Ethylene Oxide)-Based Polymer Electrolytes. Electrochim. Acta.

[65] Flory P. J. (1947). Thermodynamics
of Crystallization in High Polymers. I. Crystallization Induced by
Stretching. J. Chem. Phys..

[66] Jiang S., Zhang L., Xie T., Lin Y., Zhang H., Xu Y., Weng W., Dai L. (2013). Mechanoresponsive
PS-PnBA-PS Triblock
Copolymers via Covalently Embedding Mechanophore. ACS Macro Lett..

[67] García-Calvo J., López-Andarias J., Maillard J., Mercier V., Roffay C., Roux A., Fürstenberg A., Sakai N., Matile S. (2022). HydroFlipper Membrane Tension Probes:
Imaging Membrane Hydration and Mechanical Compression Simultaneously
in Living Cells. Chem. Sci..

[68] Humeniuk H. V., Rosspeintner A., Licari G., Kilin V., Bonacina L., Vauthey E., Sakai N., Matile S. (2018). White-Fluorescent Dual-Emission
Mechanosensitive Membrane Probes That Function by Bending Rather than
Twisting. Angew. Chem. Int. Ed..

[69] Roffay C., García-Arcos J. M., Chapuis P., López-Andarias J., Schneider F., Colom A., Tomba C., Di Meglio I., Barrett K., Dunsing V., Matile S., Roux A., Mercier V. (2024). Tutorial: Fluorescence Lifetime Microscopy of Membrane
Mechanosensitive Flipper Probes. Nat. Protoc..

[70] Pandzic, E. ; Whan, R. ; Macmillan, A. Rapid FLIM Measurement of Membrane Tension Probe Flipper-TR. In Membrane Lipids: Methods and Protocols; Cranfield, C. G. , Ed.; Springer US: New York, NY, 2022; pp 257–283. 10.1007/978-1-0716-1843-1_20 34854050

[71] Wang Z.-H., Combs C., Zhao W., Xu H. (2024). A Protocol for Measuring
Plasma Membrane Tension in the *Drosophila* Ovary Using
Fluorescence Lifetime Imaging Microscopy. STAR
Protoc..

