# Supplementary material for: Planarization of Twisted Push–Pull Probes by Stretching Rather than by Compression: Core-Substituted Fluorescent Flippers as Materials Mechanosensors
Source: JACS Au. 2025 Aug 5;5(8):3944–50. doi: 10.1021/jacsau.5c00579 (PMC12381726; doi:10.1021/jacsau.5c00579)
Supplement: Supplementary file 1 [file au5c00579_si_001.pdf]

Supporting Information

Planarization of Twisted Push-Pull Probes by Stretching Rather than by Compression: Core-Substituted Fluorescent Flippers as Materials Mechanosensors

Khurnia Krisna Puji Pamungkas,[‡⊥+ǁ] Riku Yamamoto,[§ǁ] Maxime Vonesch,[‡†] Naomi Sakai,[‡⊥] Yoshimitsu Sagara[§¶]* and Stefan Matile[‡⊥]*

[‡] Department of Organic Chemistry, University of Geneva, 1211 Geneva, Switzerland

[⊥] National Centre of Competence in Research (NCCR) Molecular Systems Engineering, 4002 Basel, Switzerland

[§] Department of Materials Science and Engineering, Institute of Science Tokyo, 2-12-1 Ookayama, Meguro-ku, Tokyo 152-8550, Japan

[¶]Research Center for Autonomous Systems Materialogy (ASMat), Institute of Science Tokyo, 4259 Nagatsuta-cho, Midori-ku, Yokohama, Kanagawa 226-8501, Japan

[+] Current address: Universitas Gadjah Mada, Sleman, Yogyakarta 55281, Indonesia

[†] Current address: Chemspeed Technologies AG, Wölferstrasse 8, 4414 Füllinsdorf, Switzerland

[ǁ] These two authors contributed equally

sagara@mct.isct.ac.jp, stefan.matile@unige.ch

Table of Contents

1.	Materials and Methods	S3
2.	Flipper Synthesis	S5
3.	Polymer Synthesis	S15
4.	Polymer Characterization	S17
5.	NMR Spectra	S24
6.	References	S37

1. Materials and Methods

As in reference S1. In brief, reagents for synthesis were purchased from Merck, TCI, Across and Alfa Aesar. Salts of the best grade available from Merck were used as received. Analytical thin layer chromatography (TLC) was performed on silica gel 60 F254 (Merck, 0.2 mm) and visualized under a UV lamp at 254 nm. Preparative TLC purifications were performed on silica gel 60 F254 (Merck, 0.25 mm) or GF (SiliCycle, 1 mm). Column chromatography was carried out on silica gel 60 (SilicaFlash® P60, SILICYCLE, 230-400 mesh).

Melting points (Mp) were measured on a Melting Point M-565 (BUCHI). IR spectra were recorded on a Perkin Elmer Spectrum 100 or Spectrum Two FT-IR spectrometer (ATR, Golden Gate) and are reported as wavenumbers v in cm^{-1} with band intensities indicated as br (broad), s (strong), m (medium), w (weak). All ^{1}H and ^{13}C NMR spectra were recorded (as indicated) on a Bruker 300 MHz, 400 MHz or 500 MHz spectrometer at rt (25 °C) and are reported as chemical shifts (δ) in parts per million (ppm) with reference to the residual solvent peak ($CDCl_3$: 7.26/77.1 ppm; CD_2Cl_2: 5.32/53.8 ppm). Spin multiplicities are reported as singlet (s), doublet (d), and triplet (t) with coupling constants (J) given in Hz, or multiplet (m). Broad peaks are marked as br. ^{1}H and ^{13}C resonances were assigned with the aid of additional information from 1D and 2D NMR spectra (H,H-COSY, DEPT 135, HSQC and HMBC). ESI-MS was measured using Advion expression CMS. ESI-HRMS was measured on Xevo G2-S Tof (Waters). All mass data are reported as mass-per-charge ratio m/z (intensity in %, [assignment]).

For polymer preparation, inhibitor-free anhydrous THF (FUJIFILM Wako Pure Chemical Corporation) was used as the solvent. Hydroxy-terminated poly(tetrahydrofuran) (PTHF) (M_n = 2,000) was dried in vacuo at 100 ºC over molecular sieves for 12 h before polymerization. 4,4'-Methylenebis(phenyl isocyanate) (MDI) and 1,4-butanediol (BDO) were distilled under reduced pressure and stored over molecular sieves in the fridge and at rt,

respectively. Molecular weights of polymers were measured by a SHIMADZU Nexera GPC system equipped with a GPC KF-805L column (ID = 8.0 mm, L = 300 mm, particle size = 10 μm). Samples were injected using THF as the eluent at 40 °C and a flow rate was 1.0 mL min^{-1}. Data was evaluated on LabSolutions software (SHIMADZU) and molecular weights were calculated based on standard polystyrene calibration (1,100−2,500,000). Differential scanning calorimetry (DSC) measurements were performed with a Hitachi DSC7020 under N_2 at heating and cooling rates of 10 °C/min. Thermogravimetric analyses (TGA) were also performed under N_2 with a SHIMADZU DTG-60. The heating rate was 10 °C/min. Stress-strain measurements were conducted under ambient conditions with a SHIMADZU AGS-100NX equipped with a 100 N load cell at a strain rate of 0.2 s^{-1}. UV-vis absorption spectra were measured on a JASCO V-750. Steady-state fluorescence spectra of polyurethane films were monitored with an Ocean Insight QEPro-FL equipped with a Reflection/Backscattering Probe R400-7-UV-VIS. These spectra were not corrected. The excitation light at 490 nm was obtained by passing the light of an Asahi Spectra CL-1501 equipped with an Asahi Spectra CL-H1-505-9-1 through a bandpass filter (Asahi Spectra HMX490）. The excitation light at 365 nm was obtained using an Ocean Insight LDC-1 equipped with Ocean Insight LSM-365A LED light source through a bandpass filter (Asahi Spectra HMZ365）. Photographs were taken with a Canon EOS 9000D stabilized with a tripod. Time-resolved fluorescence measurements were carried out with a Hamamatsu Photonics Quantaurus-Tau.

Abbreviations. AcOH: Acetic acid; DMF: Dimethylformamide; DDQ: 2,3-Dichloro-5,6-dicyano-1,4-benzoquinone; IR: Infrared; LDA: Lithium diisopropylamide; *m*-CPBA: meta-Chlorobenzoic acid; Mp: Melting point; NaAsc: Sodium ascorbate; NBS: *N*-Bromosuccinimid; pTLC: Preparative thin layer chromatography; rt: room temperature; TBTA: Tris((1-benzyl-4-triazolyl)methyl)amine; pTsOH: *p*-Toluenesulfonic acid; THF: Tetrahydrofuran; TLC: Thin layer chromatography.

2. Synthesis

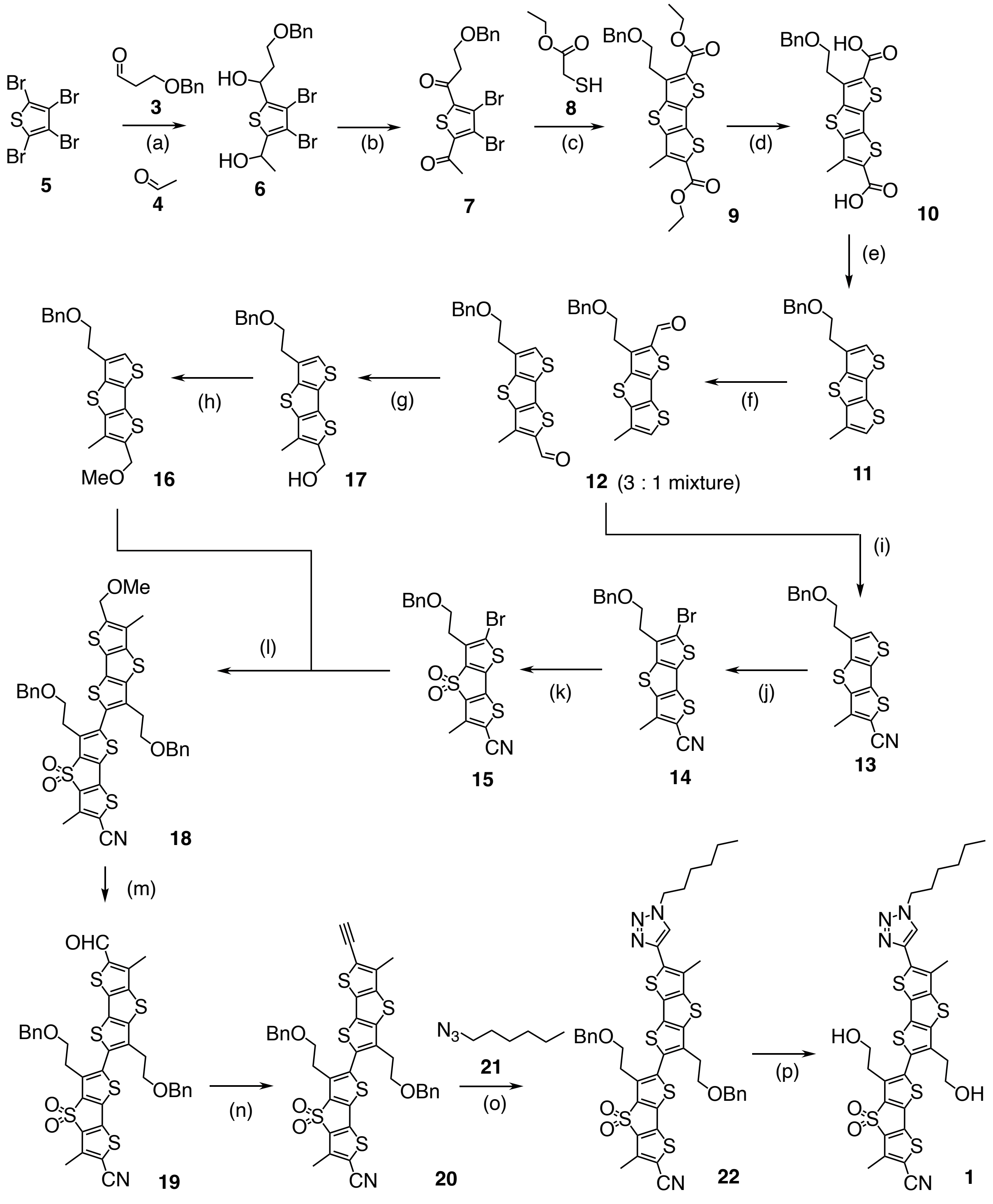

Scheme S1. (a) *n*-BuLi, **3**, **4**, THF, -78 to 0 °C to rt, Ar, 18 h; (b) Dess-Martin periodinane, CH_2Cl_2, rt, 1 h, 2 steps 56%; (c) K_2CO_3, **8**, EtOH, reflux, Ar, 3 h, 78%; (d) KOH, EtOH, reflux, Ar, 18 h, 61%; (e) Ag_2CO_3, AcOH, DMSO, 120 °C, Ar, 18 h, 55%; (f) $POCl_3$, DMF, 50 °C, Ar, 3 h, 63%, mixture of regio-isomers; (g) $NaBH_4$, DMF, Ar, 80 °C, 1 h, 44%; (h) NaH, MeI,

0 °C to rt, Ar, 18 h, 61%; (i) NaN_3, pTsOH, CH_3CN, rt, Ar, 20 min, 53%; (j) NBS, DMF, 80 °C, 3 h, 68%; (k) *m*-CPBA, CH_2Cl_2, rt, 18 h, 81%; (l) LDA, *n*-Bu_3SnCl, THF, -78 °C to rt, Ar, 30 min, $Pd(PPh_3)_4$, CuI, CsF, DMF, 65 °C, 4 days, 55%; (m) DDQ, CH_2Cl_2/H_2O (5:1), rt, 1 h, 94%; (n) Bestmann-Ohira reagent, K_2CO_3, THF/MeOH (2:1), Ar, 45 °C, 46 h, 35%; (o) $CuSO_4{\cdot}5H_2O$, NaAsc, TBTA, **21**, $THF/CH_2Cl_2/H_2O$ (3:3:1), rt, 1 h, 70%; (p) BBr_3, CH_2Cl_2, -78 °C to rt, 1.5 h, 75%.

Compound 3 was prepared according to the reported procedure.[S2]

Compound 6. To a solution of **5** (19 g, 47 mmol) in dry THF (200 mL) under Ar atmosphere, *n*-BuLi (1.6 M in THF, 36 mL, 57 mmol) was added dropwise at -78 °C. After subsequent dropwise addition of **4** (2.7 mL, 48 mmol), the mixture was stirred for 30 min at -78 °C. Then *n*-BuLi (1.6 M in THF, 36 mL, 57 mmol) was added dropwise to the mixture, followed by the addition of the solution of **3** (11 g, 66 mmol) in dry THF (10 mL). The resulting mixture was stirred at -78 °C to rt for 18 h and quenched with sat. NH_4Cl. EtOAc (150 mL) and water (150 mL) were added, and the EtOAc layer was collected. The aqueous phase was extracted with EtOAc (3 x 50 mL). Combined organic phases were washed with water and sat. $NaHCO_3$, dried over Na_2SO_4, filtered, and concentrated under reduced pressure The obtained crude mixture (22.5 g) was used directly for the next step.

Compound 7. To a solution of **6** (22.5 g, 50.0 mmol) in CH_2Cl_2 (200 mL) at rt, Dess-Martin periodinane was added (47.0 g, 110 mmol) in portions. The mixture was stirred at rt for 1 h. Solutions of NaOH (1.0 M, 100 mL) and saturated $Na_2S_2O_3$ (20 mL) were added, phases were separated, and the organic layer was washed with water (3 x 50 mL) and brine (50 mL), dried over Na_2SO_4, and filtered. Solvents were evaporated in vacuo and the product was purified by chromatography on silica gel (CH_2Cl_2/*n*-Pen 1:1) to give **7** (12.4 g, 2 steps 56%) as a colorless solid. R_f (CH_2Cl_2/*n*-Pen 1:1): 0.30; Mp: 79 – 80 °C; IR (neat): 2866 (w, CH), 1651

(s, C=O), 1477 (w), 1358 (w), 1248 (m, CO), 1187 (m, CO), 1123 (w), 814 (w), 732 (w), 695 (w, C-Br); ^{1}H NMR (400 MHz, $CDCl_3$): 7.36 – 7.28 (m, 5H), 4.55 (s, 2H), 3.90 (t, $^3J_{H-H}$ = 6.2 Hz, 2H), 3.38 (t, $^3J_{H-H}$ = 6.2 Hz, 2H), 2.75 (s, 3H); ^{13}C NMR (101 MHz, $CDCl_3$): 190.3 (C), 189.8 (C), 142.6 (C), 142.3 (C), 138.0 (C), 128.6 (2CH), 127.9 (3CH), 119.5 (C), 119.2 (C), 73.6 (CH_2), 64.9 (CH_2), 42.3 (CH_2), 29.9 (CH_3).

Compound 9. To a suspension of **7** (7.5 g, 17 mmol) in EtOH (100 mL), K_2CO_3 (3.5 g, 25 mmol) and **8** (4.4 mL, 40 mmol) were added. The reaction mixture was heated at reflux under Ar atmosphere for 3 h, and a considerable amount of precipitate formed. The mixture was finally cooled to rt, and the precipitate was collected by filtration and washed with EtOH (20 mL) and water (200 mL) to afford **9** as a yellowish solid (6.4 g, 78%). Mp: 127 – 128 ºC; IR (neat): 2848 (w, CH), 1690 (s, C=O), 1510 (w), 1381 (w), 1246 (s, CO), 1106 (s, CO), 1073 (w), 1020 (w), 753 (w, CH), 698 (w, CH); ^{1}H NMR (400 MHz, $CDCl_3$): 7.32 – 7.21 (m, 7H), 4.53 (s, 2H), 4.39 (q, $^3J_{H-H}$ = 7.1 Hz, 2H), 4.36 (q, $^3J_{H-H}$ = 7.1 Hz, 2H), 3.83 (t, $^3J_{H-H}$ = 6.6 Hz, 2H), 3.50 (t, $^3J_{H-H}$ = 6.6 Hz, 2H), 2.68 (s, 3H), 1.41 (t, $^3J_{H-H}$ = 7.1 Hz, 3H), 1.38 (t, $^3J_{H-H}$ = 7.1 Hz, 3H); ^{13}C NMR (101 MHz, $CDCl_3$): 163.0 (C), 162.6 (C), 146.0 (C), 145.8 (C), 140.8 (C), 139.2 (C), 138.4 (C), 133.0 (C), 132.4 (C), 129.2 (C), 128.7 (C), 128.4 (2CH), 127.7 (2CH), 127.6 (CH), 73.0 (CH_2), 69.1 (CH_2), 61.4 (CH_2), 61.3 (CH_2), 30.0 (CH_2), 15.0 (CH_3), 14.5 (CH_3), 14.5 (CH_3).

Compound 10. To a suspension of the **9** (2.4 g, 5.0 mmol) in EtOH (50 mL), KOH (0.83 g, 15 mmol) was added. The reaction was stirred and heated at 80 ºC for 18 h at reflux under Ar atmosphere. The solution was cooled to rt, and 1.0 M HCl was added until reaching pH = 1. The formed yellow precipitate was filtered and washed with H_2O (3 x 50 mL) and EtOH (1 x 20 mL). The solid was dried under high vacuum to give **10** as a yellow solid (1.3 g, 61%). The obtained solid was used directly for the next step.

Compound 11. To a solution of compound **10** (3.2 g, 7.5 mmol) in DMSO (30 mL), silver carbonate (1.0 g, 3.7 mmol) and AcOH (0.50 mL, 8.2 mmol) were added at room temperature. The reaction mixture was stirred for 18 h at 120 °C under Ar atmosphere. The mixture was cooled to rt, diluted in EtOAc (50 mL) and extracted (3 x 50 mL). The combined organic phase was washed with brine (3 x 100 mL), dried over Na_2SO_4, and concentrated *in vacuo*. The crude product was purified by column chromatography (SiO_2, CH_2Cl_2/*n*-Pen 3:7) to afford **11** as a yellowish oil (1.4 g, 55%). R_f (CH_2Cl_2/*n*-pen 3:7): 0.28; IR (neat): 2855 (m, CH), 1437 (m), 1362 (s), 1100 (s, CO), 1028 (w), 998 (m), 731 (s, C=C), 697 (s, CH); ^{1}H NMR (400 MHz, $CDCl_3$): 7.27 – 7.19 (m, 5H), 7.00 (t, $^4J_{H\text{-}H}$ = 1.0 Hz, 1H), 6.89 (q, $^4J_{H\text{-}H}$ = 1.1 Hz, 1H), 4.50 (s, 2H), 3.78 (t, $^3J_{H\text{-}H}$ = 6.8 Hz, 2H), 3.00 (td, $^3J_{H\text{-}H}$ = 6.8, $^4J_{H\text{-}H}$ = 1.0 Hz, 2H), 2.31 (d, $^4J_{H\text{-}H}$ = 1.1 Hz, 3H); ^{13}C NMR (101 MHz, $CDCl_3$): 142.5 (C), 141.8 (C), 138.3 (C), 132.8 (C), 131.1 (C), 131.1 (C), 130.7 (C), 128.5 (2CH), 127.9 (2CH), 127.8 (CH), 121.6 (CH), 121.0 (CH), 73.2 (CH_2), 69.0 (CH_2), 30.4 (CH_2), 14.7 (CH_3).

Compound 12. To a solution of **11** (0.30 g, 1.1 mmol) in dry DMF (15 mL) under Ar atmosphere was added $POCl_3$ (0.10 mL, 1.1 mmol) dropwise at 0 °C. After addition, the solution was heated at 50 °C for 3 h (the solution turned from yellow to orange), cooled to rt, and poured into a solution of aq. sodium acetate (sat. 50 mL) and stirred for an additional 2 h. The mixture was extracted with CH_2Cl_2 (3 x 20 mL) and washed with aq. LiCl (5%, 3 x 10 mL), dried over Na_2SO_4 and concentrated under vacuum. The yellow crude product was purified by column chromatography (SiO_2, *n*-Pen/AcOEt 10:1) to afford **12** as a mixture of regioisomers (3:1) as a colorless solid (205 mg, 63%). This mixture was used for the next reaction. R_f (*n*-Pen/AcOEt 10:1): 0.20.

Compound 17. To a solution of **12** (250 mg, 670 µmol) in dry DMF (7 mL), $NaBH_4$ (122 mg, 3.20 mmol) was added at rt. The solution was stirred at 80 °C under Ar atmosphere for 1 h. During this time, the green solution turned orange. MeOH (10 mL) and water (10 mL)

were added, and the milky mixture was extracted with CH_2Cl_2 (4 x 30 mL). The combined organic phase was washed with sat. NH_4Cl (50 mL), dried over Na_2SO_4, filtered, and concentrated in vacuo. The obtained crude product was purified by pTLC (*n*-Pen/EtOAc 5:2) to afford the corresponding **17** as a colorless solid (111 mg, 44%). R_f (*n*-Pen/EtOAc 5:2): 0.37; IR (neat): 3309 (bw, OH), 2887 (m, CH), 2862 (m, CH), 1449 (m), 1348 (m), 1082 (s, CO), 1101 (s, CO), 747 (s, CH), 671 (s), 649 (s), 505 (m); ^{1}H NMR (400 MHz, $CDCl_3$): 7.35 – 7.27 (m, 5H), 7.07 (t, $^4J_{H\text{-}H}$ = 1.0 Hz, 1H), 4.85 (s, 2H), 4.56 (s, 2H), 3.84 (t, $^3J_{H\text{-}H}$ = 6.7 Hz, 2H), 3.05 (td, $^3J_{H\text{-}H}$ = 6.7, $^4J_{H\text{-}H}$ 1.0 Hz, 2H), 2.35 (s, 3H); ^{13}C NMR (126 MHz, $CDCl_3$): 143.0 (C), 141.2 (C), 138.3 (C), 137.2 (C), 132.8 (C), 131.0 (C), 128.9 (C), 128.5 (2CH, C), 127.9 (2CH), 127.8 (CH), 121.8 (CH), 73.23 (CH_2), 69.0 (CH_2), 58.8 (CH_2), 30.4 (CH_2), 12.8 (CH_3).

Compound 16. To a solution of **17** (58 mg, 0.13 mmol) in dry THF (3 mL) was added NaH (60% in oil, 24 mg, 0.17 mmol) at 0 °C. After stirring for 30 min at 0 °C, iodomethane (12 µL, 0.19 mmol) was added, and the mixture was stirred at rt for 18 h under Ar atmosphere. The reaction was quenched with water at 0 °C and extracted with EtOAc (3 x 10 mL). The combined organic layer was dried over Na_2SO_4, filtered, and concentrated in vacuo. The residue was purified by column chromatography (SiO_2, *n*-Pen/AcOEt 5%) to give **16** (37 mg, 61%) as a yellow solid. R_f (*n*-Pen/AcOEt 19:1): 0.25; Mp: 125 – 126 °C; IR (neat): 3088 (w), 2916 (m, CH), 2854 (CH), 1450 (m), 1361 (s, CO), 1187 (s, CO), 1361 (s, CO), 1091 (s, OMe), 951 (m, C=C), 697 (CH); ^{1}H NMR (400 MHz, $CDCl_3$): 7.29 – 7.20 (m, 5H), 7.00 (d, $^4J_{H\text{-}H}$ = 1.0 Hz, 1H), 4.58 (s, 2H), 4.51 (s, 2H), 3.78 (t, $^3J_{H\text{-}H}$ = 6.7 Hz, 2H), 3.35 (s, 3H), 3.00 (td, $^3J_{H\text{-}H}$ = 6.7, $^4J_{H\text{-}H}$ = 1.0 Hz, 2H), 2.28 (s, 3H); ^{13}C NMR (101 MHz, $CDCl_3$): 142.8 (C), 141.1 (C), 138.3 (C), 134.5 (C), 132.8 (C), 131.0 (C), 129.3 (C), 129.2 (C), 128.5 (2CH), 127.9 (2CH), 127.8 (CH) 73.3 (CH_2), 69.0 (CH_2), 67.7 (CH_2), 57.9 (CH_3), 30.5 (CH_2), 12.9 (CH_3).

Compound 13. To a stirred mixture of **12** (400 mg, 1.1 mmol) and sodium azide (160 mg, 2.5 mmol) in dry MeCN (12 mL) was added pTsOH (810 mg, 5.4 mmol). The mixture was

stirred at rt until the complete consumption of **12** was evidenced by TLC (~20 min). The resulting blue solution was added to aq. $NaHSO_3$ (1.0 M, 10 mL) before being extracted with CH_2Cl_2 (2 x 20 mL). The combined organic phase was dried over Na_2SO_4 and concentrated *in vacuo*. The crude product was purified by column chromatography (SiO_2, *n*-Pen/AcOEt 15:1) to afford **13** (210 mg, 53%) as a colorless solid. R_f (*n*-Pen/AcOEt 10:1): 0.22; IR (neat): 3062 (m), 2855 (s), 2205 (s, C≡N), 1448 (s, CH), 1385 (s, CH)), 1301 (w), 1101 (s, CO), 812 (w), 740 (s, CH), 698 (s); ^{1}H NMR (400 MHz, $CDCl_3$): 7.34 – 7.28 (m, 4H), 7.23 (t, $^4J_{H-H}$ = 1.0 Hz, 1H), 4.56 (s, 2H), 3.82 (t, $^3J_{H-H}$ = 6.4 Hz, 2H), 3.05 (td, $^3J_{H-H}$ = 6.4, $^4J_{H-H}$ = 1.0 Hz, 2H), 2.55 (s, 3H); ^{13}C NMR (101 MHz, $CDCl_3$): 145.7 (C), 142.8 (C), 141.2 (C), 138.1 (C), 134.1 (C), 133.3 (C), 130.2 (C), 128.5 (2CH), 127.9 (2CH), 127.9 (CH), 125.0 (CH), 114.8 (CN), 104.5 (C), 73.3 (CH_2), 68.8 (CH_2), 30.4 (CH_2), 14.7 (CH_3).

Compound 14. To a solution of **13** (35.5 mg, 96.0 µmol) in DMF (1 mL) at 80 °C was added NBS (25.6 mg, 144 µmol). The resulting solution was stirred at 80 °C for 3 h. The orange solution was cooled to rt and diluted with brine (20 mL). The milky solution was extracted with EtOAc (3 x 15 mL). The combined organic layer was washed with sat NH_4Cl (20 mL), sat. $NaHCO_3$, water (20 mL), and brine (20 mL) and dried over Na_2SO_4. The solution was filtered, and the solvent was evaporated in vacuo. The yellow crude product was purified by crystallization (MeOH/CH_2Cl_2 1:2). The obtained crystals were collected and washed with cold MeOH to give **14** (29.5 mg, 68%) as colorless crystals. Mp: 125 – 126 °C; IR (neat): 2196 (w, CH), 2853 (w, CH), 2204 (s, C≡N), 1445 (m), 1434 (m), 1363 (s), 1110 (s, CO), 726 (s), 691 (s), 455 (C-Br); ^{1}H NMR (400 MHz, $CDCl_3$): 7.32 – 7.24 (m, 5H), 4.54 (s, 2H), 3.77 (t, $^3J_{H-H}$ = 6.5 Hz, 2H), 3.07 (t, $^3J_{H-H}$ = 6.5 Hz, 2H), 2.54 (s, 3H); ^{13}C NMR (101 MHz, $CDCl_3$): 144.5 (C), 142.7 (C), 140.5 (C), 138.1 (C), 133.4 (C), 132.8 (C), 128.9 (C), 128.5 (2CH), 127.8 (3CH), 113.6 (CN), 104.8 (CBr), 73.2 (CH_2), 68.0 (CH_2), 30.1 (CH_2), 14.7 (CH_3).

Compound 15. To a solution of **14** (33 mg, 74 μmol) in CH_2Cl_2 (0.5 mL) was added *m*-CPBA (70%, 54 mg, 0.22 mmol), and the solution was stirred at rt. The solution quickly turned bright green. After 3 h of reaction, another portion of *m*-CPBA (70%, 54 mg, 0.22 mmol) was added, and the solution was stirred at rt for 18 h. A saturated solution of $NaHCO_3$ (30 mL) was added, and layers were separated. The aqueous layer was extracted with CH_2Cl_2 (3 x 20 mL), and the combined organic phase was dried over Na_2SO_4, filtered, and concentrated in *vacuo*. The crude product was purified by column chromatography (SiO_2, CH_2Cl_2/*n*-Pen =1:1) to give **15** (29 mg, 81%) as a yellow solid. R_f (CH_2Cl_2/*n*-Pen = 1:1): 0.20; Mp: 183 – 184 °C; IR (neat): 2862 (w, CH), 2214 (m, C≡N), 1411 (w), 1309 (s, SO_2), 1146 (s, CO), 1103 (m), 698 (w, C-Br), 672 (w); ^{1}H NMR (400 MHz, $CDCl_3$): 7.33 – 7.26 (m, 5H), 4.55 (s, 1H), 3.82 (t, $^3J_{H-H}$ = 6.7 Hz, 2H), 3.08 (t, $^3J_{H-H}$ = 6.9 Hz, 2H), 2.59 (s, 3H); ^{13}C NMR (101 MHz, $CDCl_3$): 143.9 (C), 143.9 (C), 140.9 (C), 139.6 (C), 138.2 (C), 135.1 (C), 132.4 (C), 128.5 (2CH), 127.7 (2CH), 127.7 (CH), 118.5 (CBr), 112.5 (CN), 109.2 (C), 73.1 (CH_2), 68.3 (CH_2), 28.6 (CH_2), 13.2 (CH_3).

Compound 18. To a solution of **16** (203 mg, 522 μmol) in dry THF (3 mL) under Ar atmosphere at –78 °C was added LDA (2.0 M in THF/heptane/ethylbenzene, 392 μL, 784 μmol). The resulting bright green solution was stirred at –78 °C for 5 min. Bu_3SnCl (240 μL, 840 μmol) was added to give a yellow solution, which was stirred at –78 °C for 30 min and then at rt for 30 min under Ar atmosphere. The THF solution was concentrated under vacuum. Then, to the residue under Ar atmosphere at rt were added **15** (250 mg, 522 μmol), CsF (160 mg, 1.04 mmol), dry DMF (4 mL), $Pd(PPh_3)_4$ (79 mg, 68 μmol), and CuI (13 mg, 68 μmol). The reaction was stirred at 65 °C for 96 h. The red solution was diluted with water (20 mL) and extracted with CH_2Cl_2 (3 x 20 mL). The combined organic layer was washed with LiCl 5% (3 x 25 mL), dried over Na_2SO_4, filtered, and evaporated in vacuo. The obtained residue was purified by column chromatography (SiO_2, CH_2Cl_2/$CHCl_3$ 8:2) to give **18** (226

mg, 55%) as a red solid. R_f ($CH_2Cl_2/CHCl_3$ 8:2): 0.15; Mp: 102 – 103 °C; IR (Neat): 2923 (m), 2857 (m), 2212 (m, C≡N), 1453 (m), 1416 (m), 1316 (s, SO_2), 1143 (s, CO), 1097 (s), 738 (m); ^{1}H NMR (400 MHz, $CDCl_3$): 7.25 – 7.18 (m, 10H), 4.66 (s, 2H), 4.45 (s, 2H), 4.44 (s, 2H), 3.77 (t, $^3J_{H\text{-}H}$ = 7.0 Hz, 2H), 3.71 (t, $^3J_{H\text{-}H}$ = 6.6 Hz, 2H), 3.44 (s, 3H), 3.03 (t, $^3J_{H\text{-}H}$ = 7.0 Hz, 2H), 2.93 (t, $^3J_{H\text{-}H}$ = 6.6 Hz, 2H), 2.62 (s, 3H), 2.36 (s, 3H); ^{13}C NMR (101 MHz, $CDCl_3$): 144.9 (C), 143.8 (C), 143.6 (C), 141.4 (C), 141.1 (C), 140.2 (C), 138.7 (C), 138.2 (C), 138.0 (C), 136.3 (C), 135.5 (C), 134.7 (C), 133.3 (C), 132.1 (C), 129.1 (C), 128.6 (C), 128.4 (2CH), 128.4 (2CH), 127.9 (2CH), 127.9 (2CH), 127.8 (CH), 127.7 (CH), 126.2 (C), 112.6 (CN), 109.1 (C), 73.2 (CH_2), 73.1 (CH_2), 69.0 (CH_2), 68.3 (CH_2), 67.8 (CH_2), 58.1 (CH_3), 29.5 (CH_2), 28.0 (CH_2), 13.3 (CH_3), 12.9 (CH_3).

Compound 19. To a solution of **18** (29 mg, 0.037 mmol) in CH_2Cl_2 (1.5 mL) and water (0.30 mL), DDQ (34 mg, 0.15 mmol) was added, and the heterogeneous mixture was vigorously stirred at rt for 1 h. An aqueous solution of $NaHCO_3$ (saturated, 5 mL) was added, and the solution was extracted with CH_2Cl_2 (3 x 10 mL). The combined organic phase was dried over Na_2SO_4, filtered, and dried in vacuo. The crude mixture was purified using column chromatography (SiO_2, CH_2Cl_2) to afford **19** (27 mg, 94%) as an orange solid. R_f (CH_2Cl_2): 0.30; IR (Neat): 2923 (s, CH), 2857 (s, CH), 2212 (s, C≡N), 1650 (s, C=O), 1509 (w), 1416 (w), 1363 (w), 1316 (s, SO_2), 1143 (s, CO), 1097 (s, CO), 908 (w), 738 (m); ^{1}H NMR (400 MHz, $CDCl_3$): 10.13 (s, 1H), 7.24 – 7.16 (m, 10H), 4.44 (s, 2H), 4.43 (s, 2H), 3.79 (t, $^3J_{H\text{-}H}$ = 6.8 Hz, 2H), 3.69 (t, $^3J_{H\text{-}H}$ = 6.3 Hz, 2H), 3.01 (t, $^3J_{H\text{-}H}$ = 6.8 Hz, 2H), 2.92 (t, $^3J_{H\text{-}H}$ = 6.3 Hz, 2H), 2.72 (s, 3H), 2.62 (s, 3H); ^{13}C NMR (101 MHz, $CDCl_3$): 182.3 (CHO), 145.6 (C), 144.9 (C), 144.1 (C), 143.9 (C), 141.5 (C), 140.7 (C), 140.0 (C), 138.5 (C), 138.1 (C), 137.9 (C), 137.7 (C), 136.2 (C), 136.0 (C), 135.2 (C), 133.9 (C), 131.6 (C), 130.3 (C), 128.4 (4CH), 128.0 (2CH), 127.9 (2CH), 127.9 (CH), 127.8 (CH), 112.5 (C), 109.4 (CN), 73.3 (CH_2), 73.2 (CH_2), 69.0 (CH_2), 68.1 (CH_2), 29.4 (CH_2), 28.1 (CH_2), 13.5 (CH_3), 13.3 (CH_3).

Compound 20. To a solution of **19** (57 mg, 74 μmol) in THF (1 mL) under Ar atmosphere were added K_2CO_3 (72 mg, 0.52 mmol), MeOH (0.5 mL), and dimethyl (1-diazo-2-oxopropyl)phosphonate (95%, 56 μL, 354 μmol). The solution was stirred at 45 °C under Ar atmosphere for 46 h. The mixture was evaporated in vacuo to give a red crude residue, which was then purified by pTLC (CH_2Cl_2) to give **20** as an orange solid (20 mg, 35%) along with **19** (11 mg, 18%). R_f (CH_2Cl_2): 0.42; IR (neat): 3274 (m, C≡H), 2922 (s, CH), 2857 (s, CH), 2212 (m, C≡N), 2093 (w), 1723 (w), 1411 (m), 1316 (s, SO_2), 1143 (s, CO), 1100 (s, CO), 1021 (m), 910 (w), 812 (w); ^{1}H NMR (400 MHz, $CDCl_3$): 7.27 – 7.17 (m, 10H), 4.44 (s, 2H), 4.43 (s, 2H), 3.77 (t, $^3J_{H-H}$ = 6.9 Hz, 2H), 3.69 (t, $^3J_{H-H}$ = 6.5 Hz, 2H), 3.64 (s, 1H), 3.02 (t, $^3J_{H-H}$ = 6.9 Hz, 2H), 2.91 (t, $^3J_{H-H}$ = 6.5 Hz, 2H), 2.62 (s, 3H), 2.45 (s, 3H); ^{13}C NMR (126 MHz, $CDCl_3$): 144.9 (C), 143.8 (C), 143.1 (C), 142.2 (C), 141.4 (C), 140.1 (C), 138.4 (C), 138.2 (C), 138.0 (C), 137.0 (C), 135.7 (C), 134.8 (C), 133.5 (C), 131.7 (C), 129.9 (C), 129.4 (C), 128.4 (2CH), 128.4 (2CH), 128.0 (2CH), 127.9 (2CH), 127.8 (CH), 127.7 (CH), 127.2 (C), 118.8 (-**C**≡C-H), 112.6 (CN), 109.2 (C), 85.4 (-C≡**C**-H), 73.2 (CH_2), 73.1 (CH_2), 69.0 (CH_2), 68.2 (CH_2), 29.4 (CH_2), 28.1 (CH_2), 14.1 (CH_3), 13.3 (CH_3).

Compound 21 was prepared according to the reported procedure.[S3]

Compound 22. To a suspension of TBTA (5.93 mg, 11.2 μmol) in THF (300 μL) were added successively aqueous solutions of $CuSO_4.5H_2O$ (0.35 M, 30 μL, 11 μmol) and sodium ascorbate (0.38 M, 30 μL, 11 μmol). The resulting colorless solution (360 μL) was added to a mixture of **20** (4.3 mg, 5.6 μmol) and **21** (4.5 μL, 34 μmol) in CH_2Cl_2 (300 μL). The reaction mixture was stirred at rt for 1 h. A saturated solution of NH_4Cl (10 mL) was added, and the mixture was extracted with CH_2Cl_2 (3 × 10 mL), dried over Na_2SO_4, filtered, and the solvent was removed in vacuo. The crude product was purified by pTLC (CH_2Cl_2/MeOH 99:1) to afford **22** (3.5 mg, 70%) as a red solid. R_f (CH_2Cl_2/MeOH 99:1): 0.36; IR (Neat): 2924 (s, CH), 2862 (s, CH), 2341 (w), 2212 (m, C≡N), 1450 (w), 1420 (s), 1315 (s, SO_2), 1142 (s), 1100 (s,

CO), 741 (s), 700 (m), 559 (w); ^{1}H NMR (400 MHz, $CDCl_3$): 7.71 (s, 1H), 7.25 – 7.19 (m, 10H), 4.45 (s, 2H), 4.44 (s, 2H), 4.43 (t, $^3J_{H\text{-}H}$ = 7.3 Hz, 2H), 3.78 (t, $^3J_{H\text{-}H}$ = 7.0 Hz, 2H), 3.72 (t, $^3J_{H\text{-}H}$ = 6.5 Hz, 2H), 3.04 (t, $^3J_{H\text{-}H}$ = 7.0 Hz, 2H), 2.94 (t, $^3J_{H\text{-}H}$ = 6.5 Hz, 2H), 2.62 (s, 3H), 2.55 (s, 3H), 1.99 (p, $^3J_{H\text{-}H}$ = 7.3 Hz, 2H), 1.42 – 1.33 (m, 6H), 0.92 – 0.89 (m, 3H); ^{13}C NMR (101 MHz, $CDCl_3$): 144.9 (C), 144.6 (C), 143.8 (C), 142.5 (C), 141.4 (C), 141.4 (C), 140.2 (C), 138.7 (C), 138.2 (C), 138.0 (C), 135.6 (C), 134.7 (C), 133.4 (C), 132.1 (C), 129.0 (C), 128.4 (2CH), 128.4 (2CH), 128.3 (C), 127.9 (2CH), 127.9 (2CH), 127.8 (CH), 127.7 (CH), 127.6 (C), 126.5 (C), 120.1 (CH, triazole), 112.6 (CN), 109.1 (C), 73.2 (CH_2), 73.1 (CH_2), 69.0 (CH_2), 68.3 (CH_2), 50.8 (CH_2), 31.3 (CH_2), 30.5 (CH_2), 29.5 (CH_2), 28.1 (CH_2), 26.4 (CH_2), 22.6 (CH_2), 14.6 (CH_3), 14.1 (CH_3), 13.3 (CH_3).

Compound 1. A solution of **22** (15 mg, 17 μmol) in dry CH_2Cl_2 (1.5 mL) was cooled to -78 °C under Ar atmosphere. Boron tribromide (1.0 M in CH_2Cl_2, 38 μL, 38 μmol) was added dropwise to the solution and stirred for 1 h at the same temperature. Another portion of Boron tribromide (1.0 M in CH_2Cl_2, 38 μL, 38 μmol) was added to the solution and stirred for 30 min. The solution was warmed to rt, quenched with saturated aqueous $NaHCO_3$ (5 mL), extracted with CH_2Cl_2 (3 x 10 mL), dried over Na_2SO_4, filtered, and concentrated in *vacuo*. The obtained crude was purified by pTLC (CH_2Cl_2/MeOH 95:5) to give **1** (9 mg, 75%) as a red solid. R_f (CH_2Cl_2/MeOH 95:5): 0.36; IR (Neat): 3370 (bw, OH), 2926 (s, CH), 2861 (s, CH), 2213 (s, C≡N), 1716 (w, triazole), 1414 (s), 1314 (SO_2), 1142 (CO), 1050 (s), 801 (m) 557 (m); ^{1}H NMR (400 MHz, $CDCl_3$): 7.68 (s, 1H), 4.43 (t, $^3J_{H\text{-}H}$ = 7.3 Hz, 2H), 4.02 (t, $^3J_{H\text{-}H}$ = 6.3 Hz, 2H), 4.01 (t, $^3J_{H\text{-}H}$ = 6.4 Hz, 2H), 3.08 (t, $^3J_{H\text{-}H}$ = 6.4 Hz, 2H), 3.04 (t, $^3J_{H\text{-}H}$ = 6.3 Hz, 2H), 2.62 (s, 3H), 2.57 (s, 3H), 1.99 (p, $^3J_{H\text{-}H}$ = 7.4 Hz, 2H), 1.42 – 1.34 (m, 6H), 0.93 – 0.90 (m, 3H); ^{13}C NMR (101 MHz, $CDCl_3$): 145.0 (C), 144.5 (C), 143.8 (C), 142.3 (C), 141.2 (C), 141.1 (C), 140.2 (C), 138.7 (C), 135.9 (C), 134.6 (C), 133.5 (C), 132.3 (C), 129.2 (C), 128.4 (C), 127.6 (C), 126.8 (C), 120.2 (CH, triazole), 112.5 (CN), 109.4 (C), 61.7 (CH_2), 60.9 (CH_2),

50.8 (CH_2), 32.0 (CH_2), 31.3 (CH_2), 30.6 (CH_2), 30.5 (CH_2), 26.3 (CH_2), 22.6 (CH_2), 14.6 (CH_3), 14.1 (CH_3), 13.3 (CH_3); HRMS (ESI): calcd. for $C_{31}H_{31}N_4O_4S_6$ ($[M+H]^+$): 715.0665, found: 715.0628.

3. Polymer Synthesis

Polyurethane 26. Dibutyltin dilaurate (4 drops) was added to a stirred mixture of **1** (2.14 mg, 3.0×10^{-3} mmol), PTHF (M_n = 2,000, 3.00 g, 1.50 mmol), and MDI (1.26 g, 5.03 mmol) in THF (20 mL) and the mixture was stirred at rt. for 3 h. A solution of BDO (297 mg, 3.30 mmol) in THF (10 mL) was then added and the reaction mixture was additionally stirred at rt for 24 h. After MeOH (10 mL) was added to the reaction mixture, the solution was stirred for another 30 min, and then poured into MeOH (1,000 mL). The yellow precipitate was collected by filtration and dissolved in THF (150 mL). The resulting solution was filtrated through a cotton filter, and the polymer was precipitated into hexane (1,000 mL). The precipitate was filtered off and dried in vacuo for 24 h at rt to afford **26** as a yellow rubbery solid (4.0 g, 88%, M_n = 67,000, $Đ$ = 2.30).

Polyurethane 27. Dibutyltin dilaurate (4 drops) was added to a stirred mixture of PTHF (M_n = 2,000, 3.00 g, 1.50 mmol), and MDI (1.26 g, 5.03 mmol) in THF (20 mL) and the mixture was stirred at rt for 3 h. A solution of BDO (297 mg, 3.30 mmol) in THF (10 mL) was then added and the reaction mixture was additionally stirred at rt for 24 h. After MeOH (10 mL) was added to the reaction mixture, the solution was stirred for another 30 min, and then poured into MeOH (1,000 mL). The white precipitate was collected by filtration and dissolved in THF (150 mL). The resulting solution was filtrated through a cotton filter, and the polymer was precipitated into hexane (1,000 mL). The precipitate was filtered off and dried in vacuo for 24 h at rt to afford **27** as a white rubbery solid (3.9 g, 86%, M_n = 51,000, $Đ$ = 1.96).

Preparation of Polyurethane Films

Preparation of 26 films. The polyurethane **26** (300 mg) was dissolved in THF (10 mL) and the solutions were poured onto poly(tetrafluoroethylene) molds (35 × 70 × 4 mm). The solutions covered with an inverted funnel were evaporated under ambient conditions for 12 h and in vacuo at rt for 6 h. The resulting films were smooth and slightly opaque. The thickness of the films was 75–95 μm.

Preparation of flipper-doped films. **1** (141 μg), **22** (176 μg) or **28** (113 μg) was dissolved in THF solution (10 mL) of **27** (300 mg) and the solutions were poured onto poly(tetrafluoroethylene) molds (35 × 70 × 4 mm). The solutions covered with an inverted funnel were evaporated under ambient conditions for 12 h and in vacuo at rt for 6 h. The resulting films were smooth and slightly opaque. The thickness of the films was 75–95 μm.

4. Polymer Characterization

^1H NMR spectra of the polyurethanes

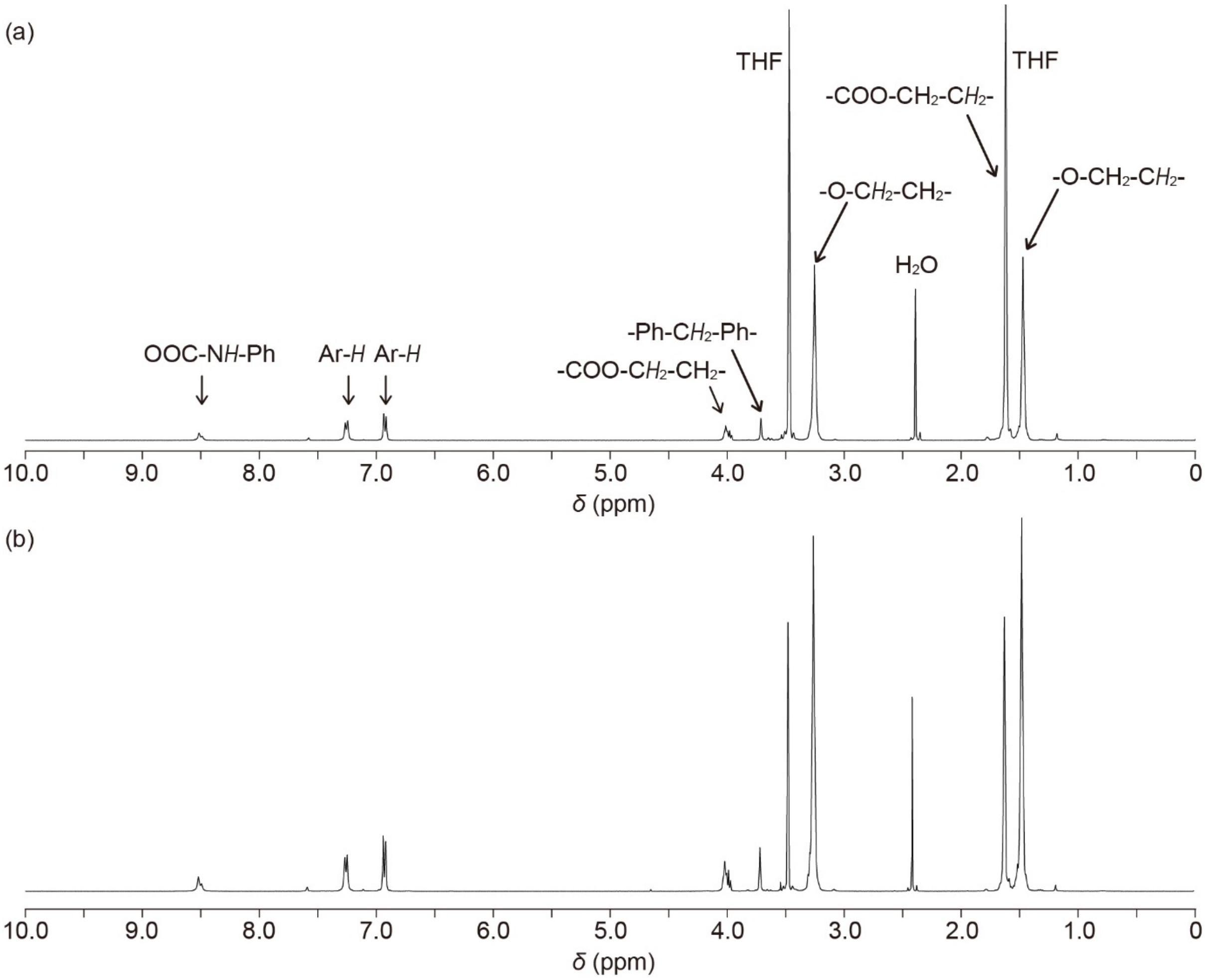

Figure S1. ^1H NMR spectra of (a) **26** and (b) **27** in THF-d_8. Signals were characterized as protons of the polyurethane chains. No signals ascribed to the residues of the flipper were observed due to their tiny quantities in **26**. All spectra were measured at rt.

Absorption spectra of polyurethane 26

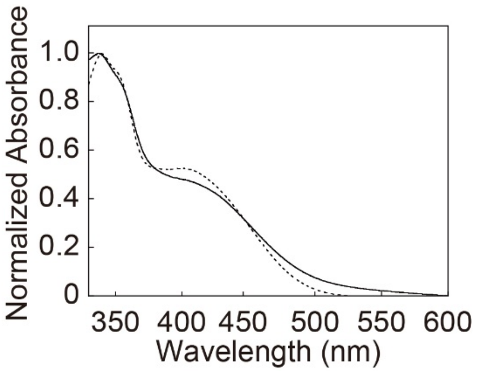

Figure S2. UV-vis absorption spectra of **26** (c = 30 mg/mL, solid line) and **1** ($c = 1.0 \times 10^{-5}$M, dotted line) in THF. The spectra were normalized at the maximum intensity.

Thermal properties of the polyurethanes

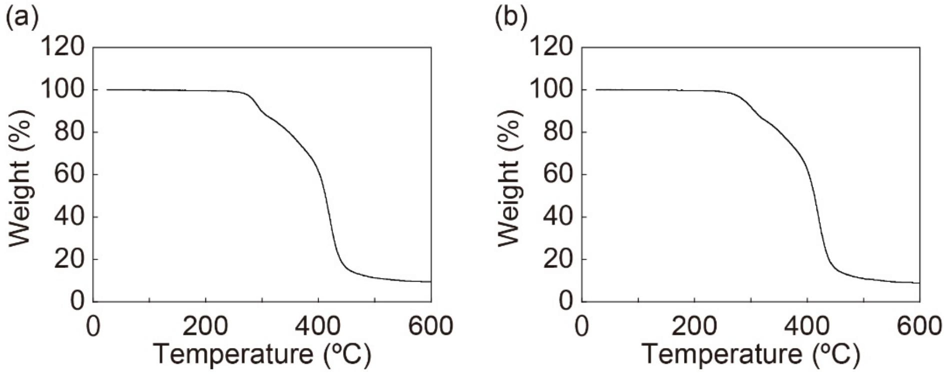

Figure S3. TGA traces of (a) **26** and (b) **27**. The heating rate was 10 °C/min.

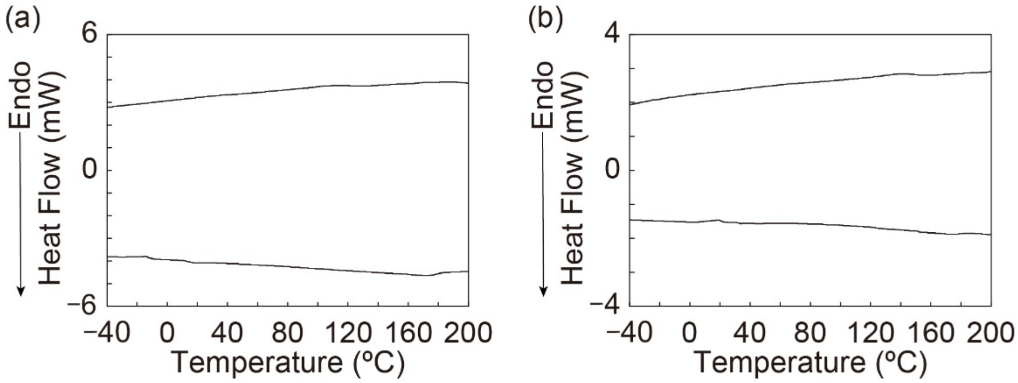

Figure S4. DSC traces of (a) **26** and (b) **27**. Shown are the first cooling and second heating curves. The heating and cooling rates were 10 °C/min.

Mechanical properties of the polyurethane films

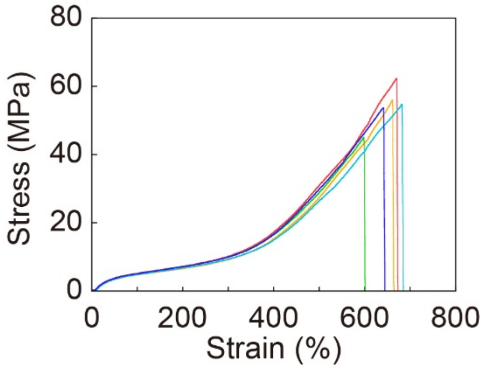

Figure S5. Stress-strain curves of **26** films. The tensile tests were carried out at a strain rate of 5 mm/s.

Table S1. Mechanical data of the polyurethane films extracted from the tensile test data.[a)]

	Elongation at break (%)	Stress at break (MPa)	Young's modulus[b)] (MPa)
**26**	651 ± 30	54.4 ± 5.6	10.1 ± 2.6

[a)] All data were extracted from the stress-strain curves shown in Figure S5 and represent averages of 5 measurements ± standard deviation. [b)]The Young's moduli were derived from the slopes of the stress-strain curves in the strain regime of 5–10%.

Mechanoresponsive luminescence of films

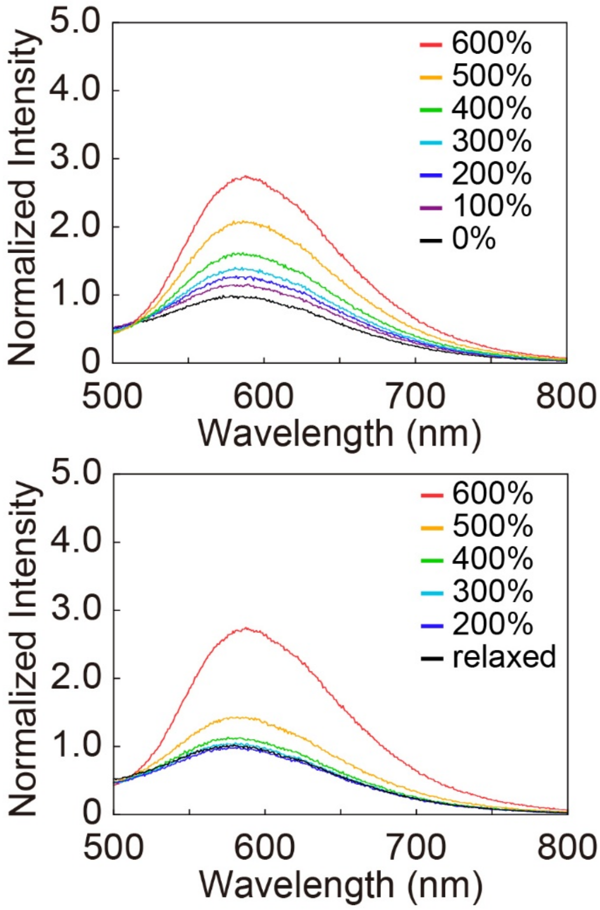

Figure S6. Emission spectra of polyurethane **26** upon stretching (top) and relaxing (bottom) the film to the indicated strains with excitation light of 365 nm.

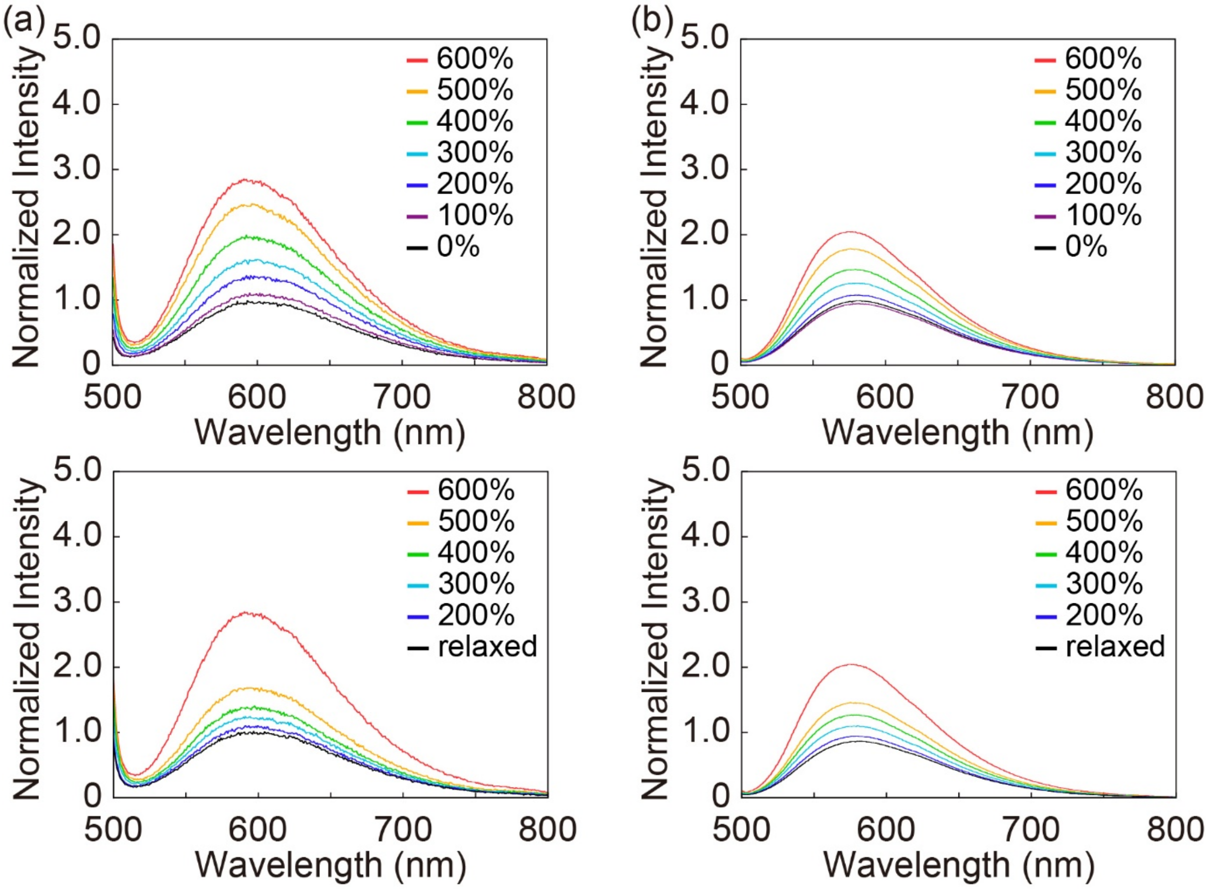

Figure S7. Emission spectra of polyurethane **27** doped with (a) **1** and (b) **22** upon stretching (top) and relaxing (bottom) the film to the indicated strains with excitation light of 490 nm.

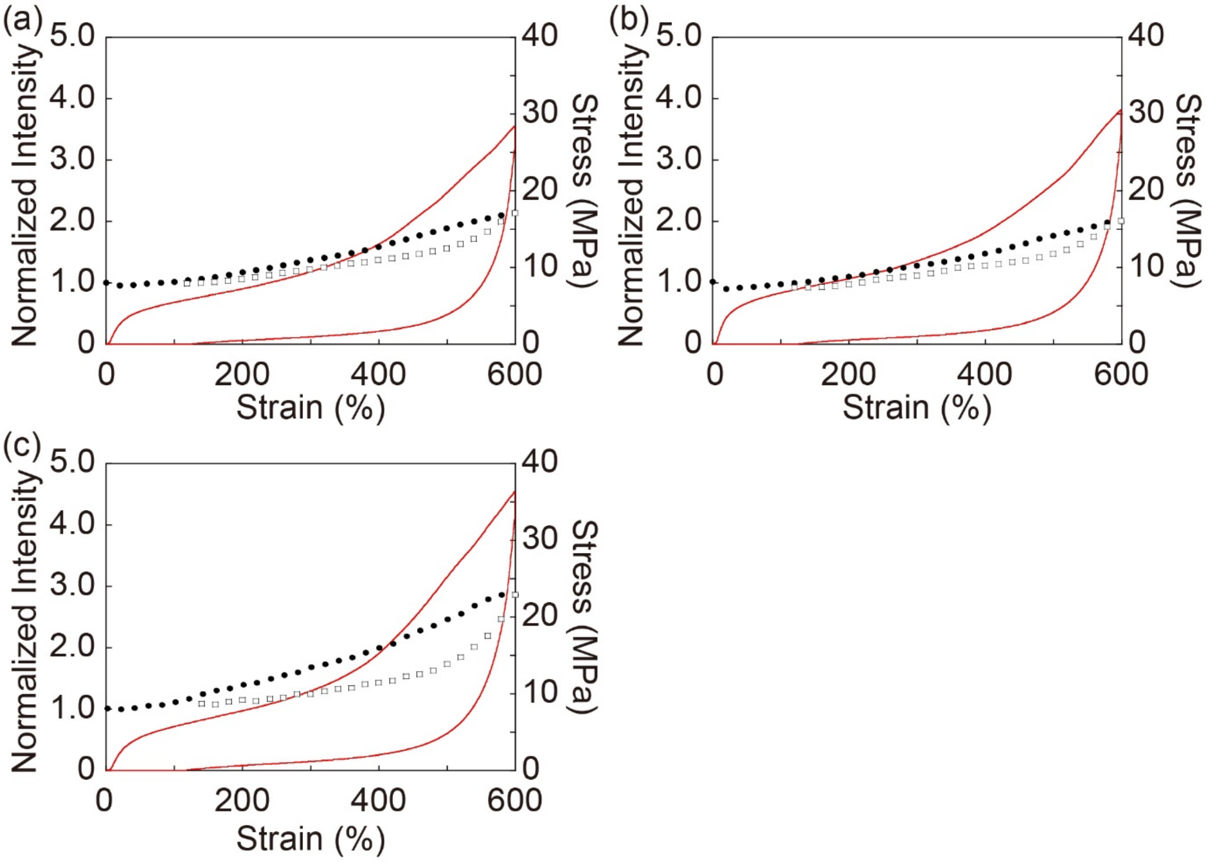

Figure S8. Overlays of the stress–strain curves (lines) and the corresponding emission intensities (circles and squares) recorded for polyurethane **27** films dope with a) **28**, b) **22**, and c) **1**. The emission intensities were recorded at 600 nm with excitation at 490 nm.

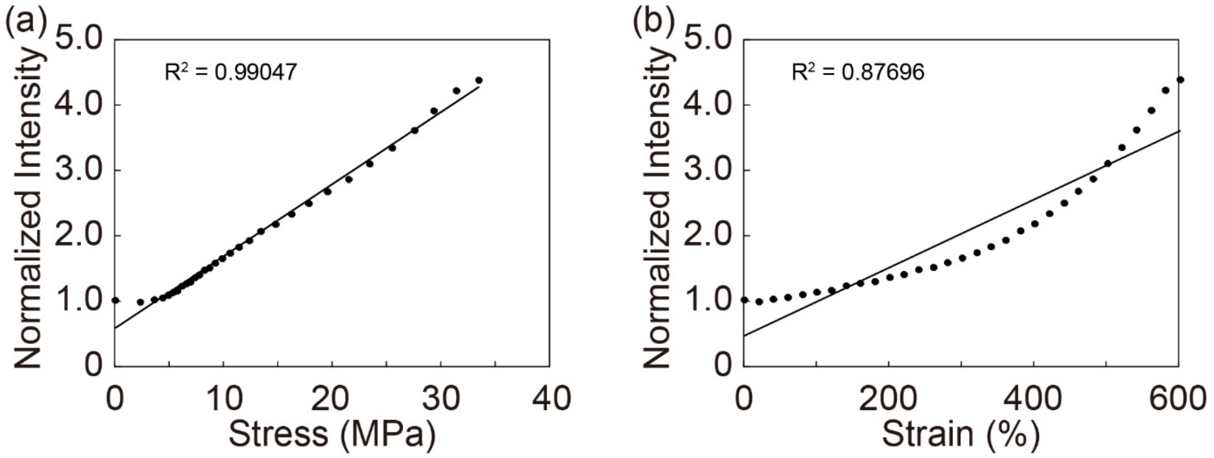

Figure S9. Plots of the normalized intensity against (a) stress and (b) strain for the first stretching **26**. Linear fits were applied to the data, and the corresponding coefficient of determinations (R^2) are shown. The data were taken from Figure 4e and re-plotted.

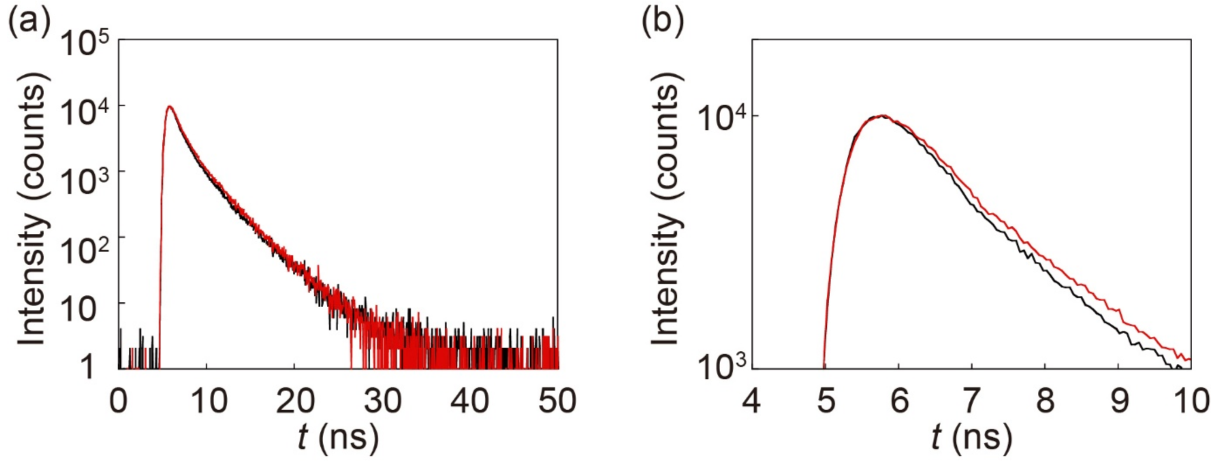

Figure S10. (a) Emission decay profiles for **26** films in the force-free state (black) and in the strain of 600% (red). The decays were monitored at 600 nm. (b) Expansion of the initial period of the decay traces shown in panel a. λ_{ex} = 470 nm.

5. NMR Spectra

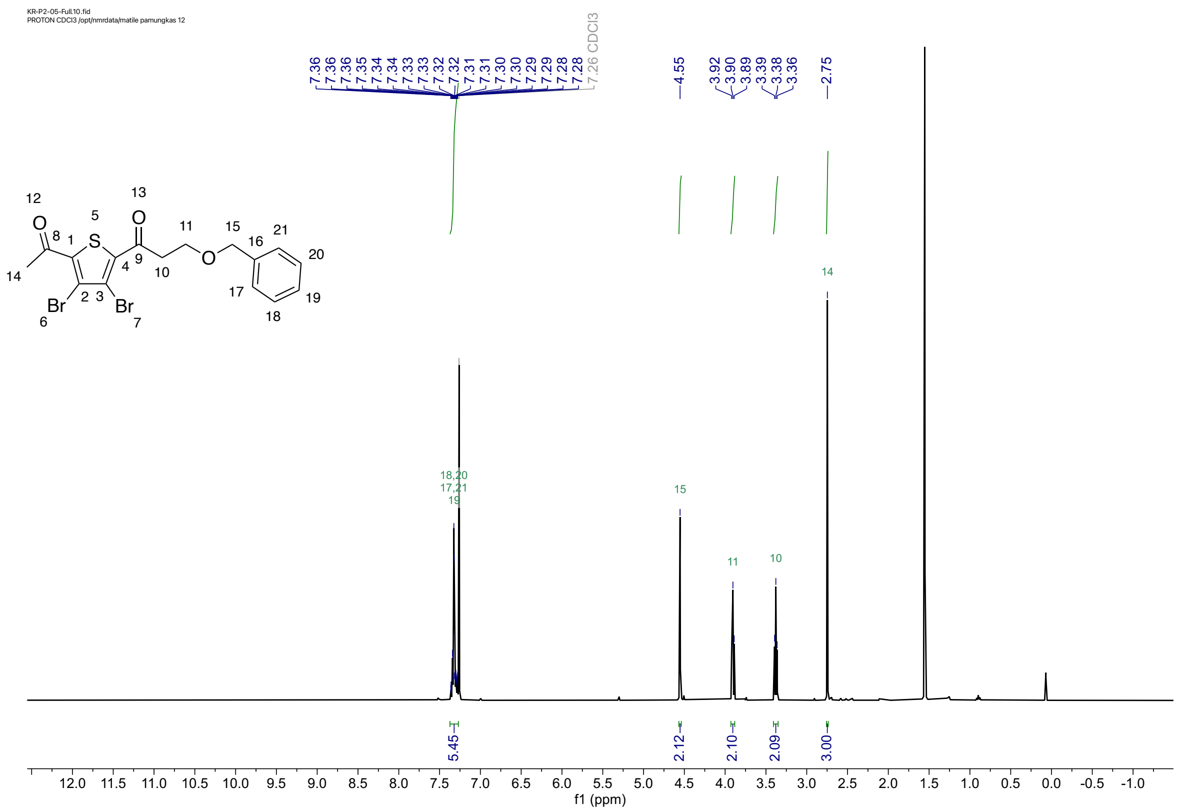

Figure S11. ^{1}H NMR (400 MHz, $CDCl_3$) spectrum of **6**.

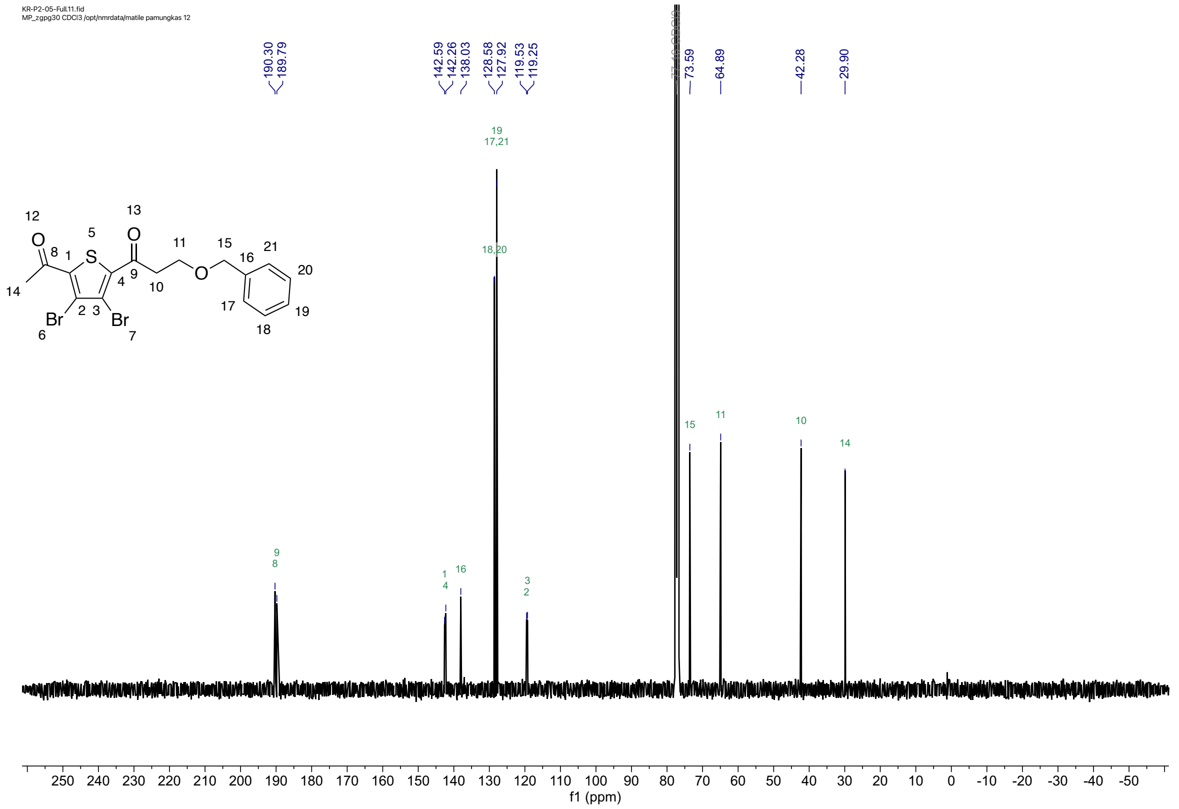

Figure S12. ^{13}C NMR (126 MHz, $CDCl_3$) spectrum of **6**.

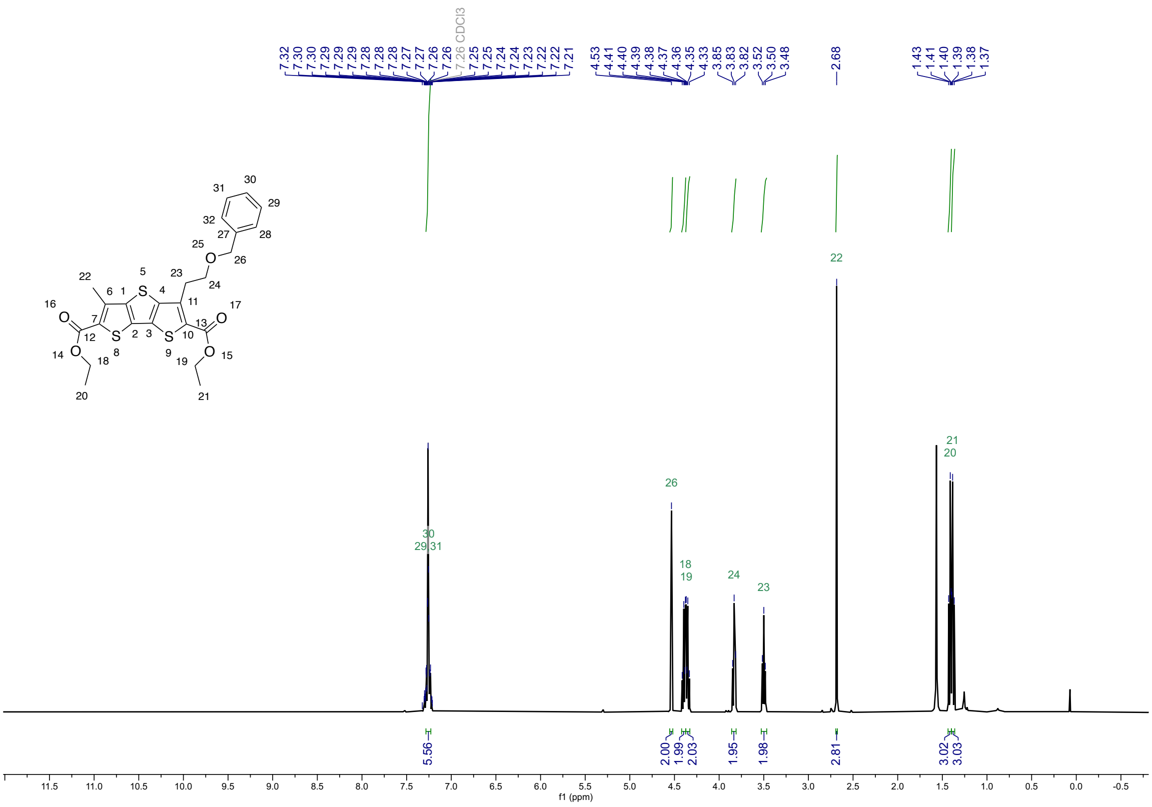

Figure S13. ^{1}H NMR (400 MHz, $CDCl_3$) spectrum of **9**.

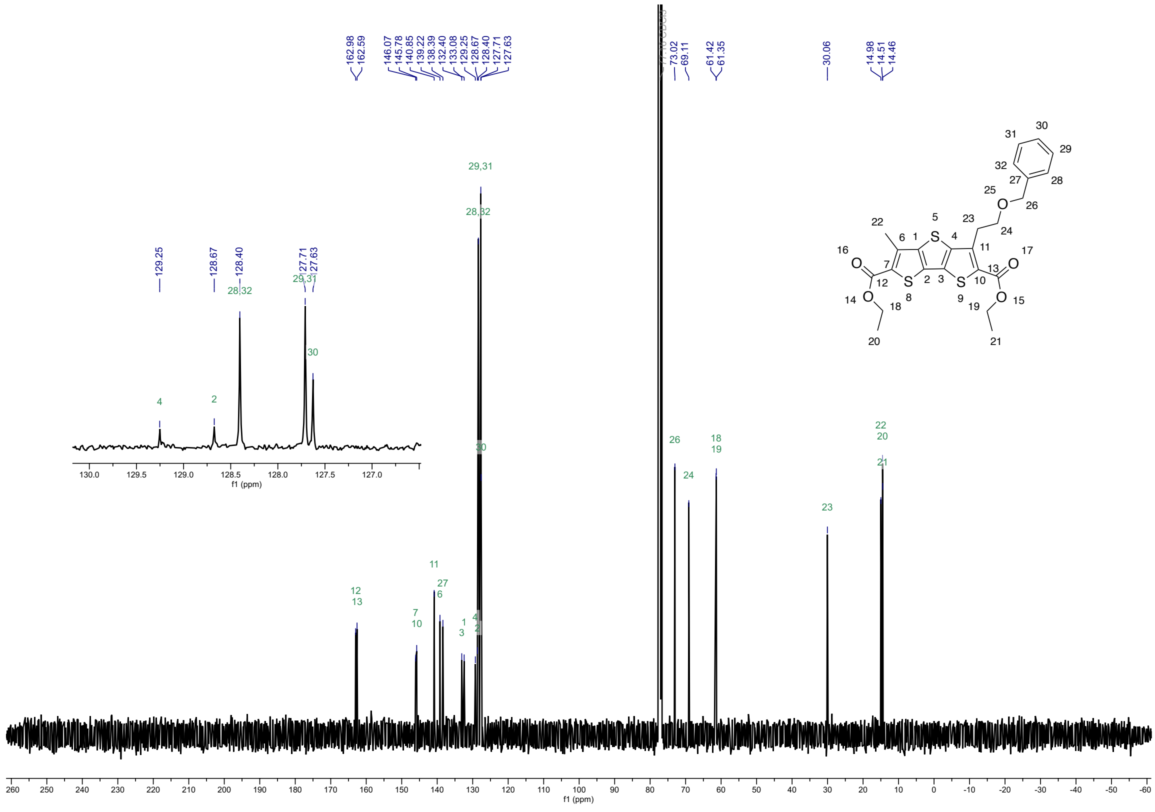

Figure S14. ^{13}C NMR (101 MHz, $CDCl_3$) spectrum of **9**.

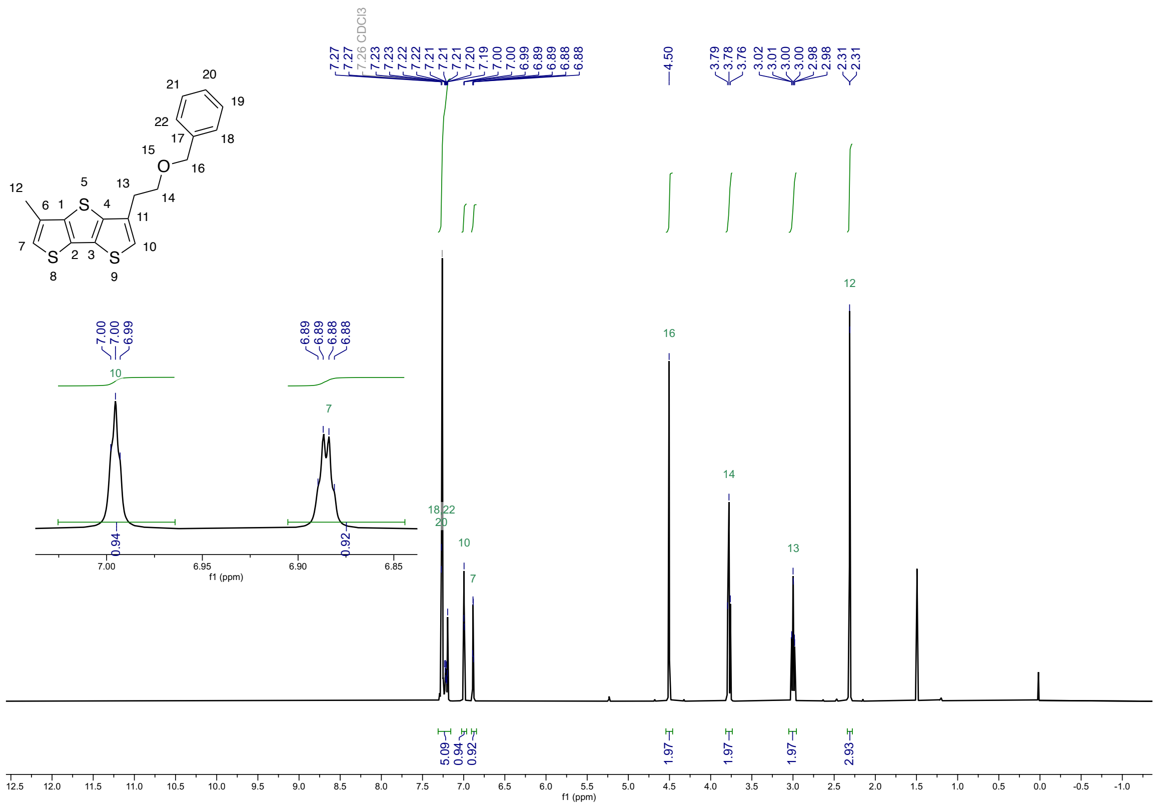

Figure S15. ^{1}H NMR (400 MHz, $CDCl_3$) spectrum of **11**.

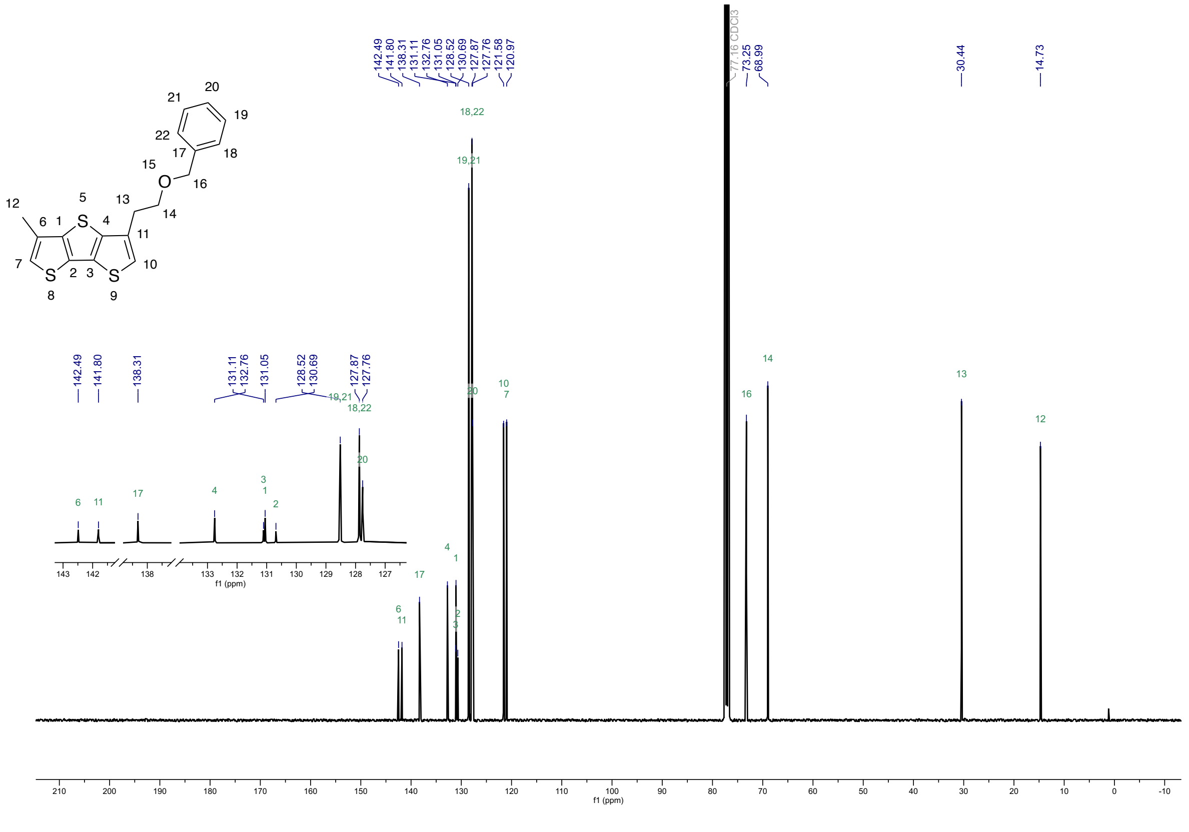

Figure S16. ^{13}C NMR (101 MHz, $CDCl_3$) spectrum of **11**.

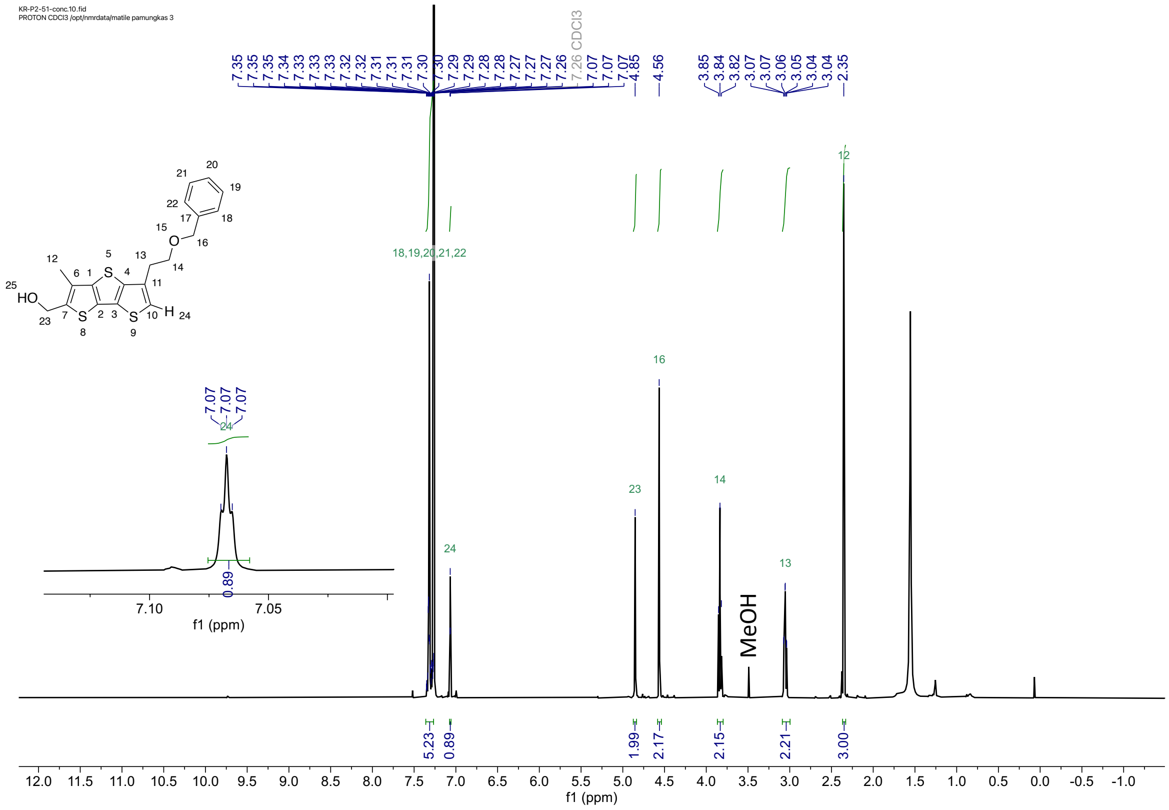

Figure S17. ^{1}H NMR (400 MHz, $CDCl_3$) spectrum of **17**.

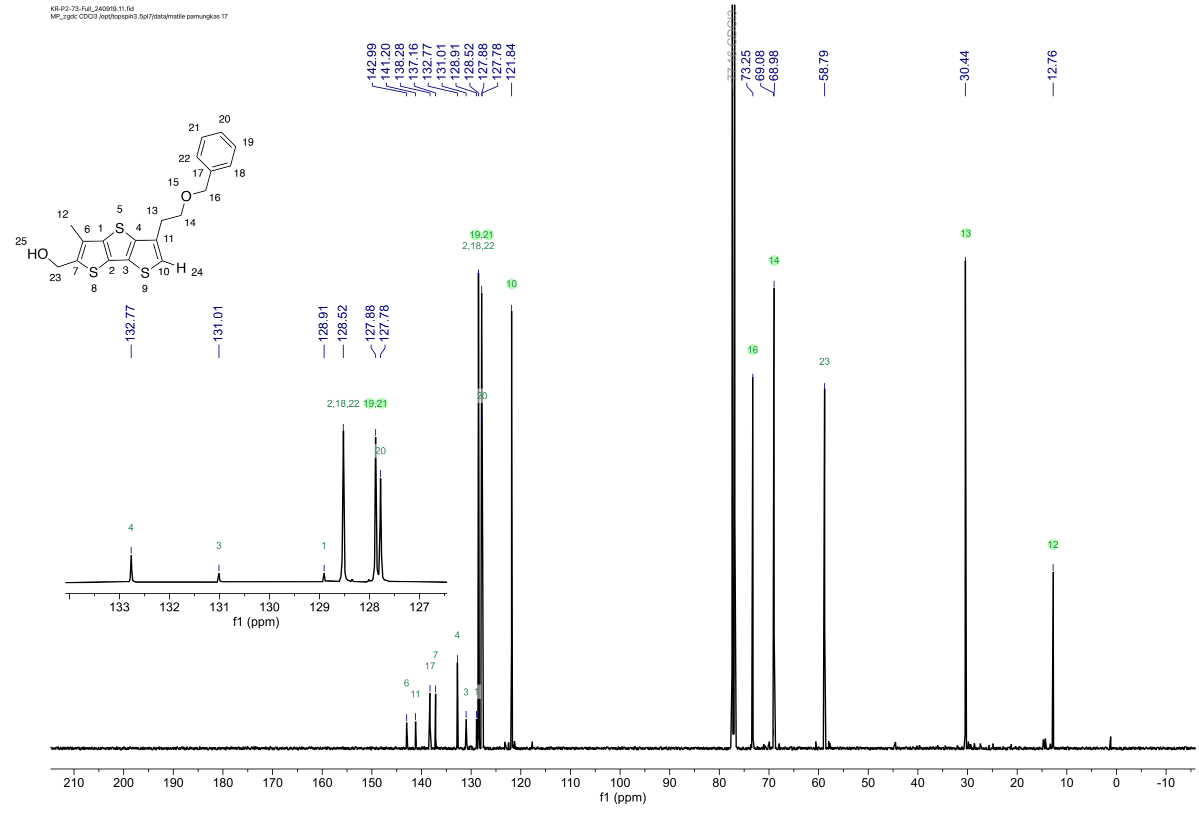

Figure S18. ^{13}C NMR (126 MHz, $CDCl_3$) spectrum of **17**.

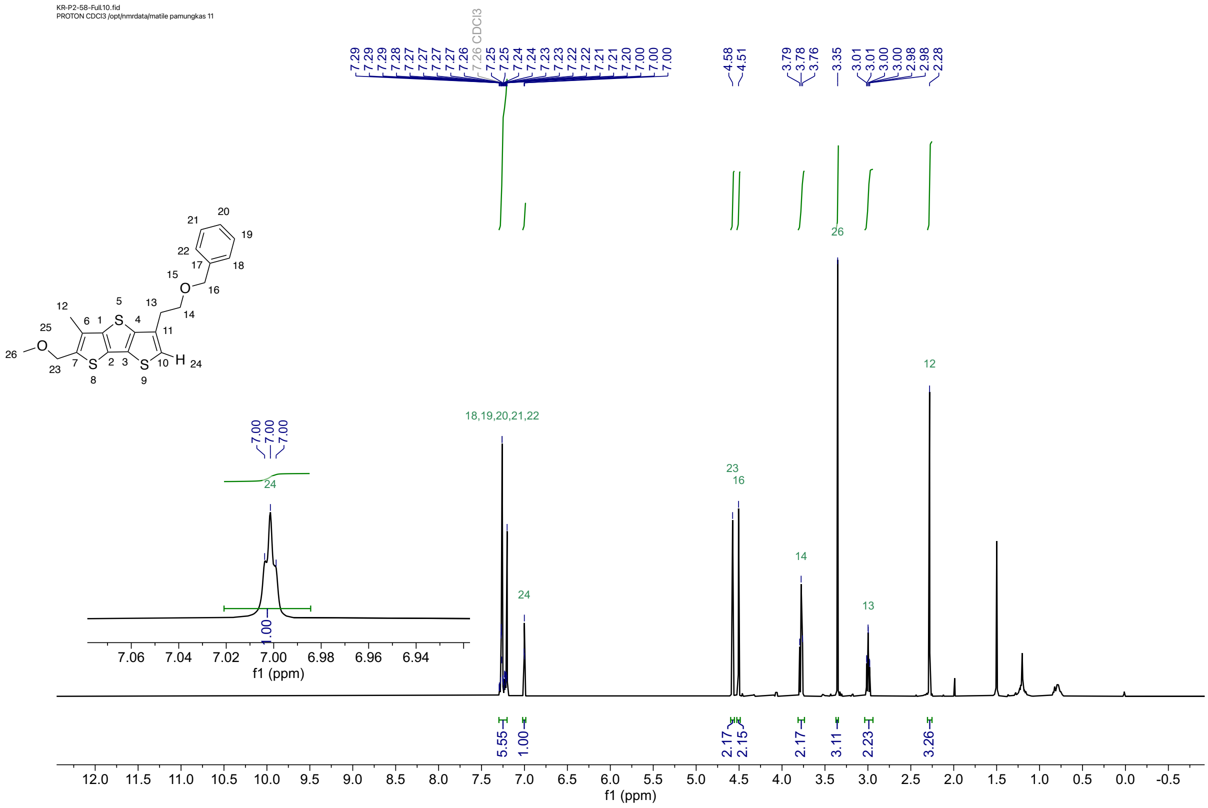

Figure S19. ^{1}H NMR (400 MHz, $CDCl_3$) spectrum of **16**.

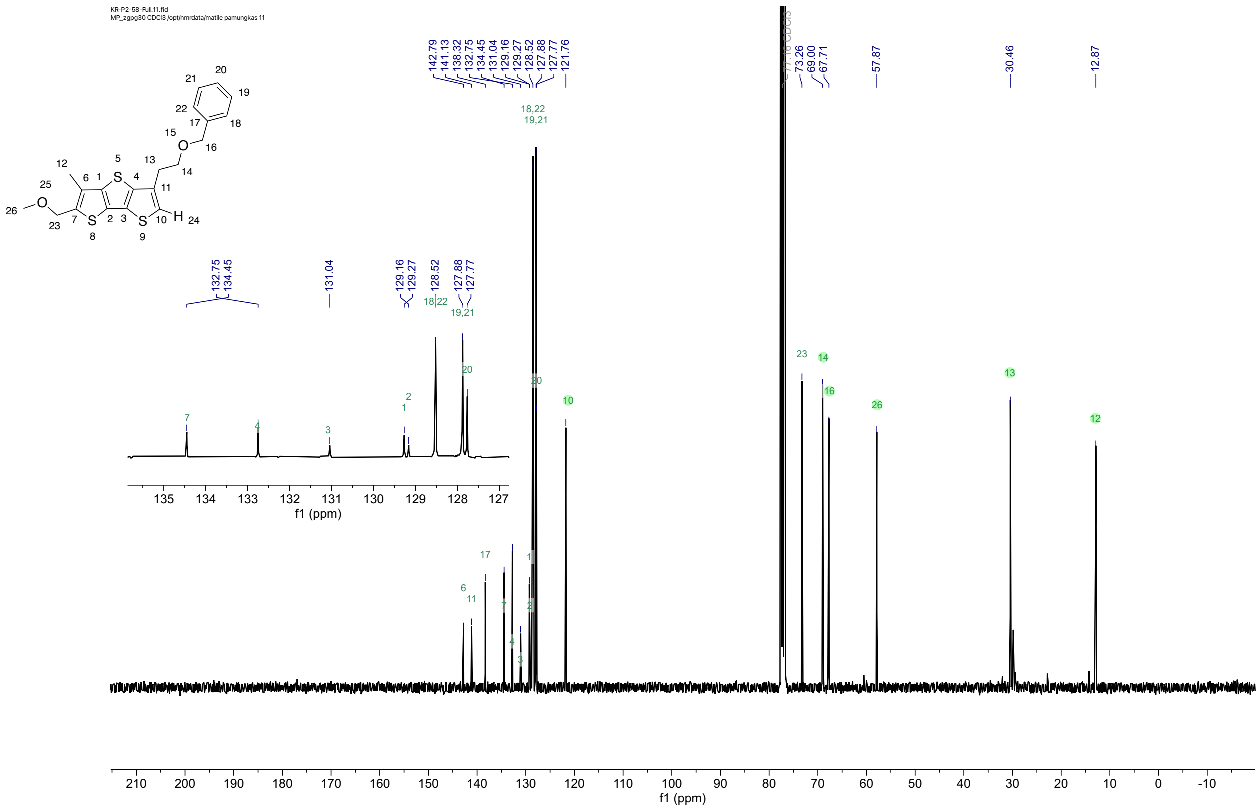

Figure S20. ^{13}C NMR (101 MHz, $CDCl_3$) spectrum of **16**.

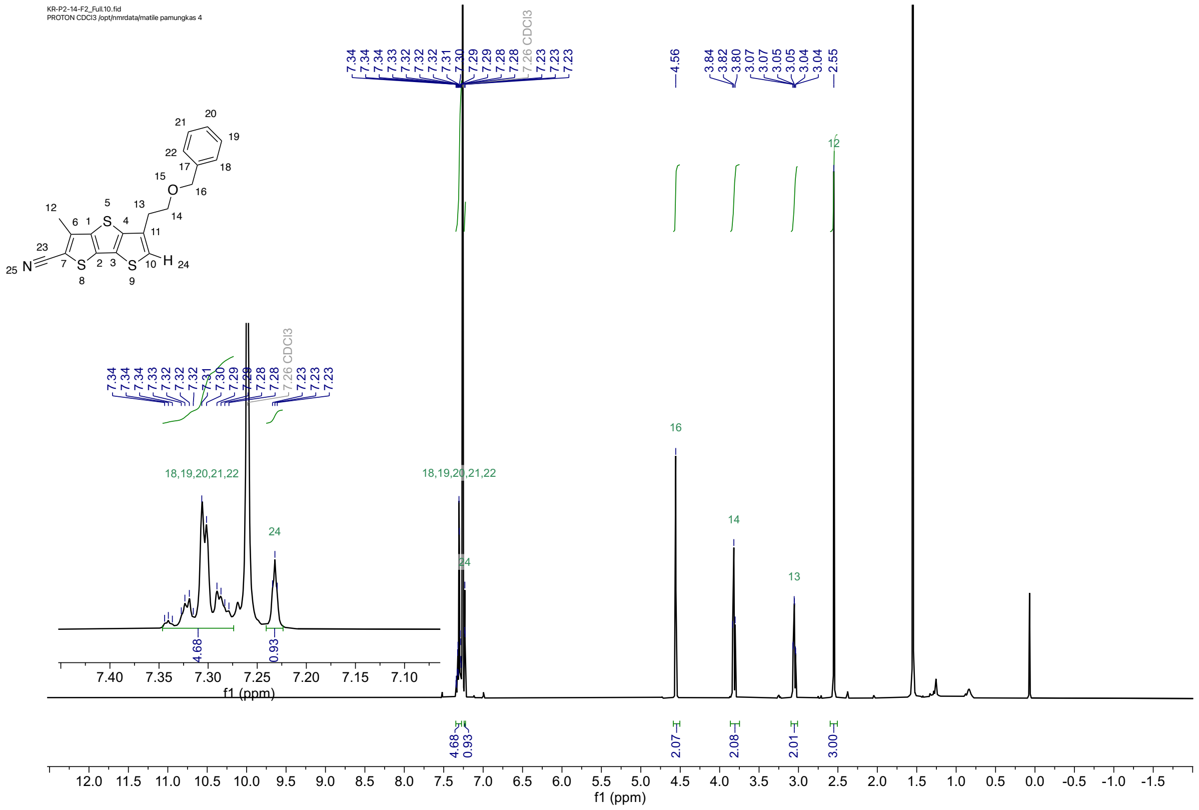

Figure S21. ^{1}H NMR (400 MHz, $CDCl_3$) spectrum of **13**.

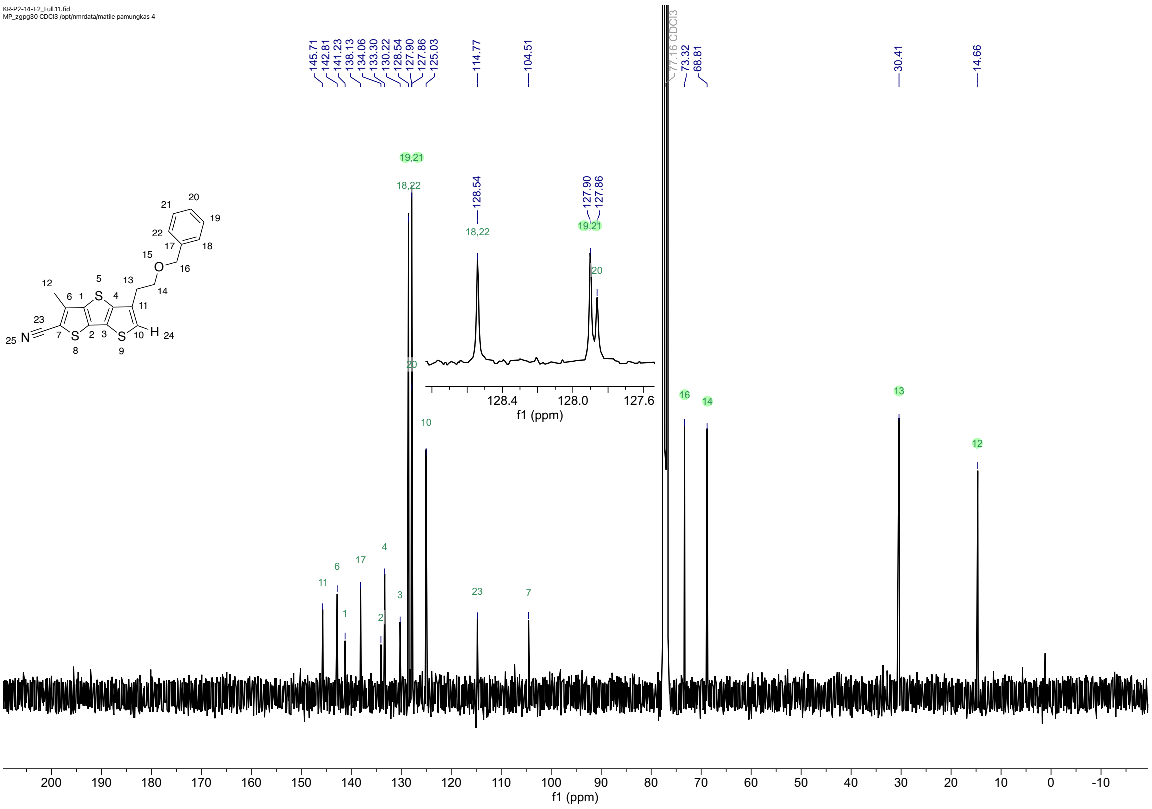

Figure S22. ^{13}C NMR (101 MHz, $CDCl_3$) spectrum of **13**.

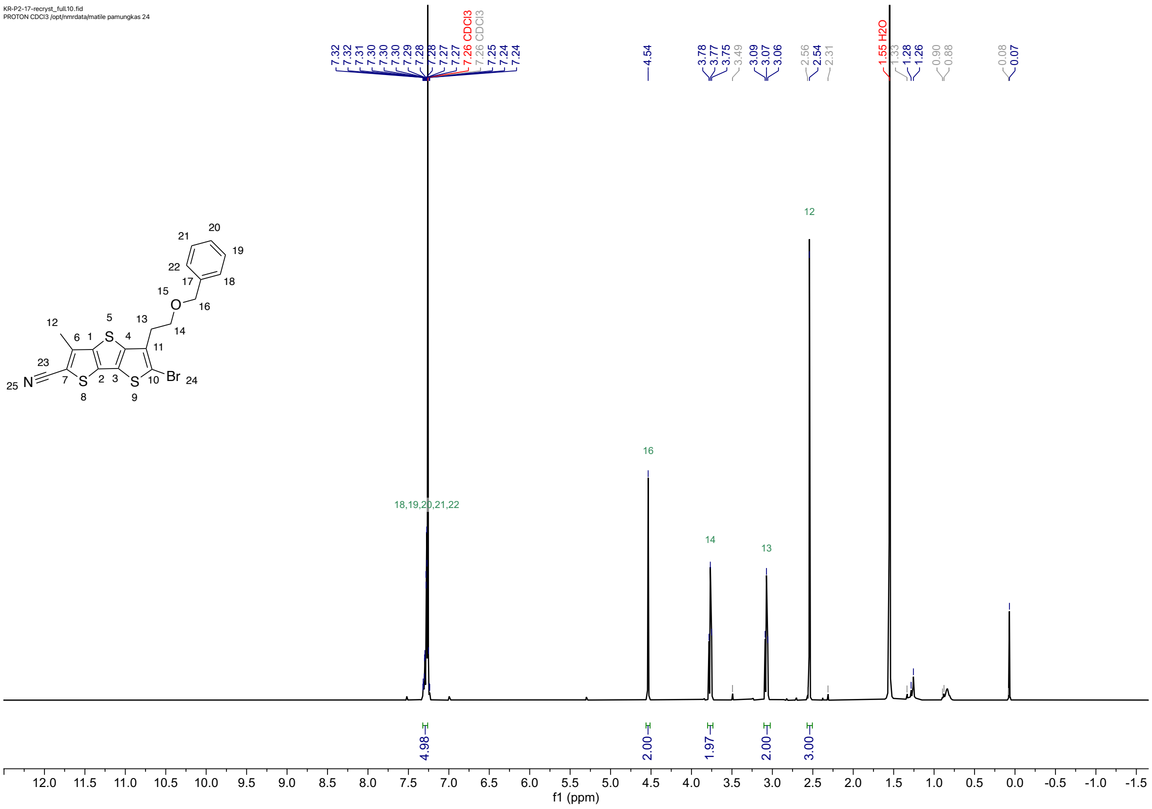

Figure S23. ^{1}H NMR (400 MHz, $CDCl_3$) spectrum of **14**.

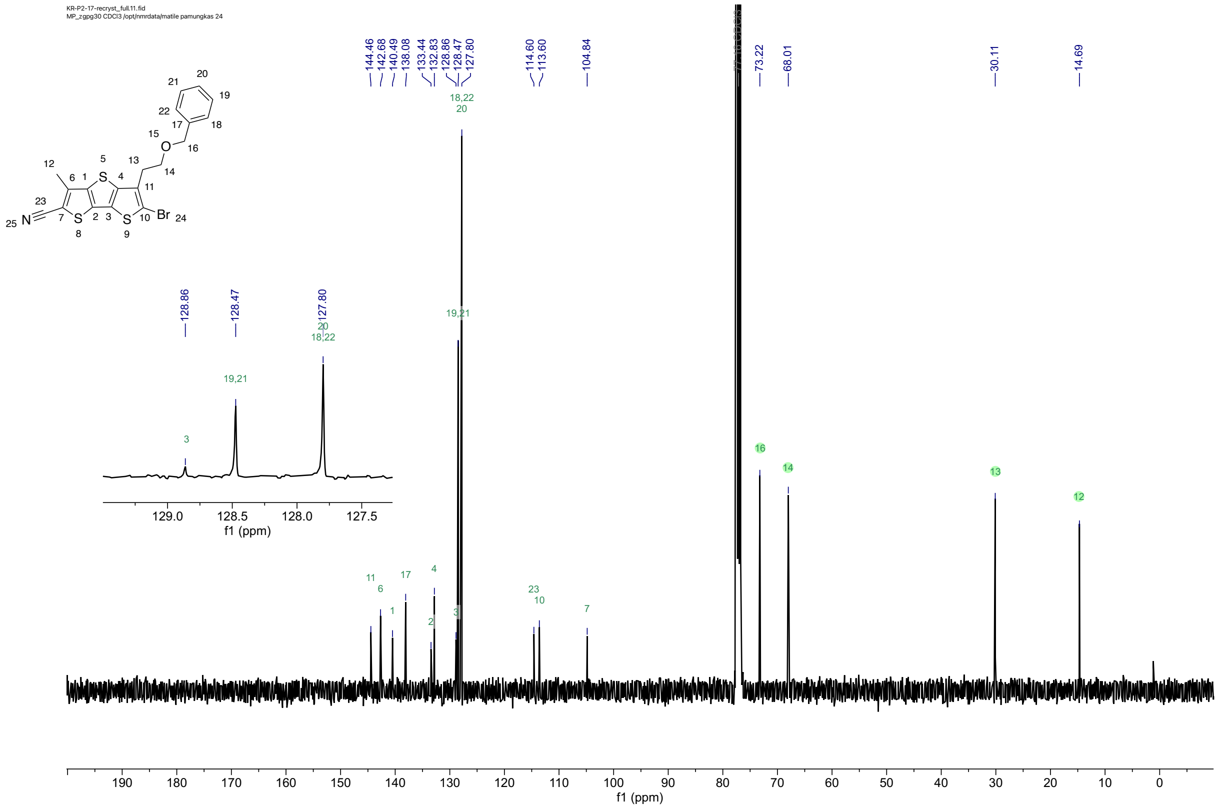

Figure S24. ^{13}C NMR (101 MHz, $CDCl_3$) spectrum of **14**.

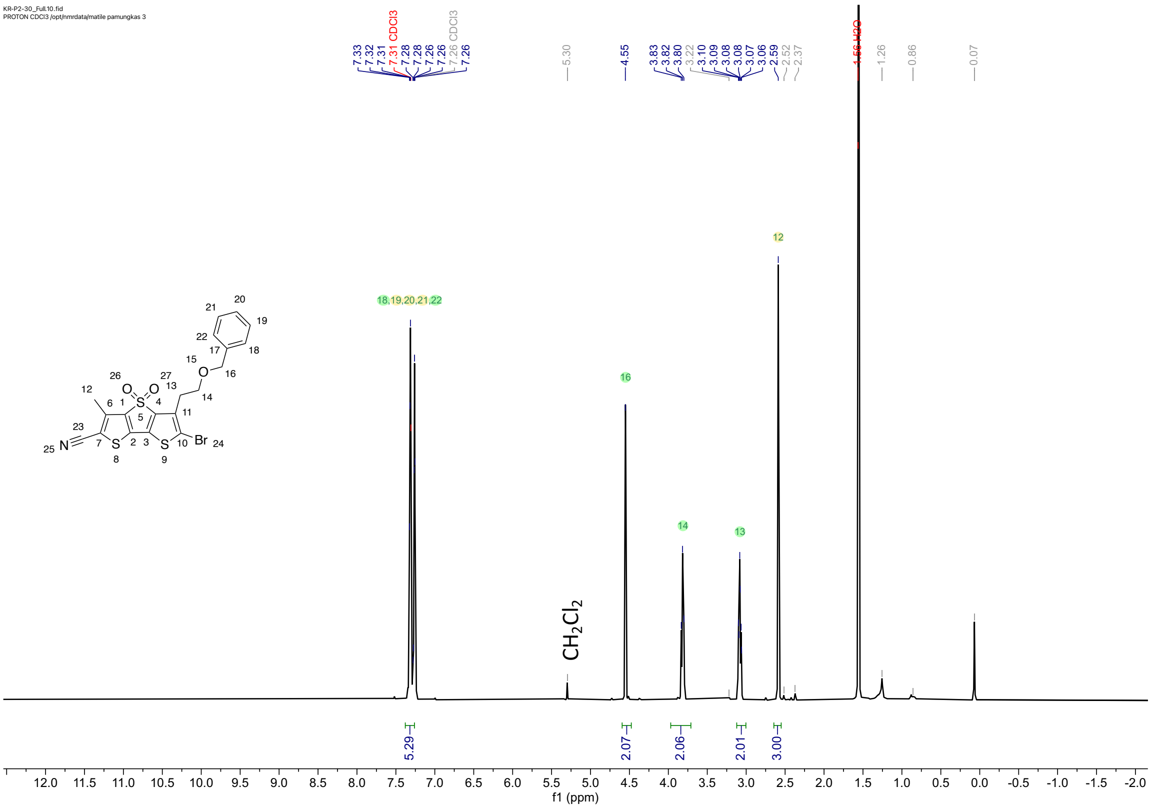

Figure S25. ^{1}H NMR (400 MHz, $CDCl_3$) spectrum of **15.**

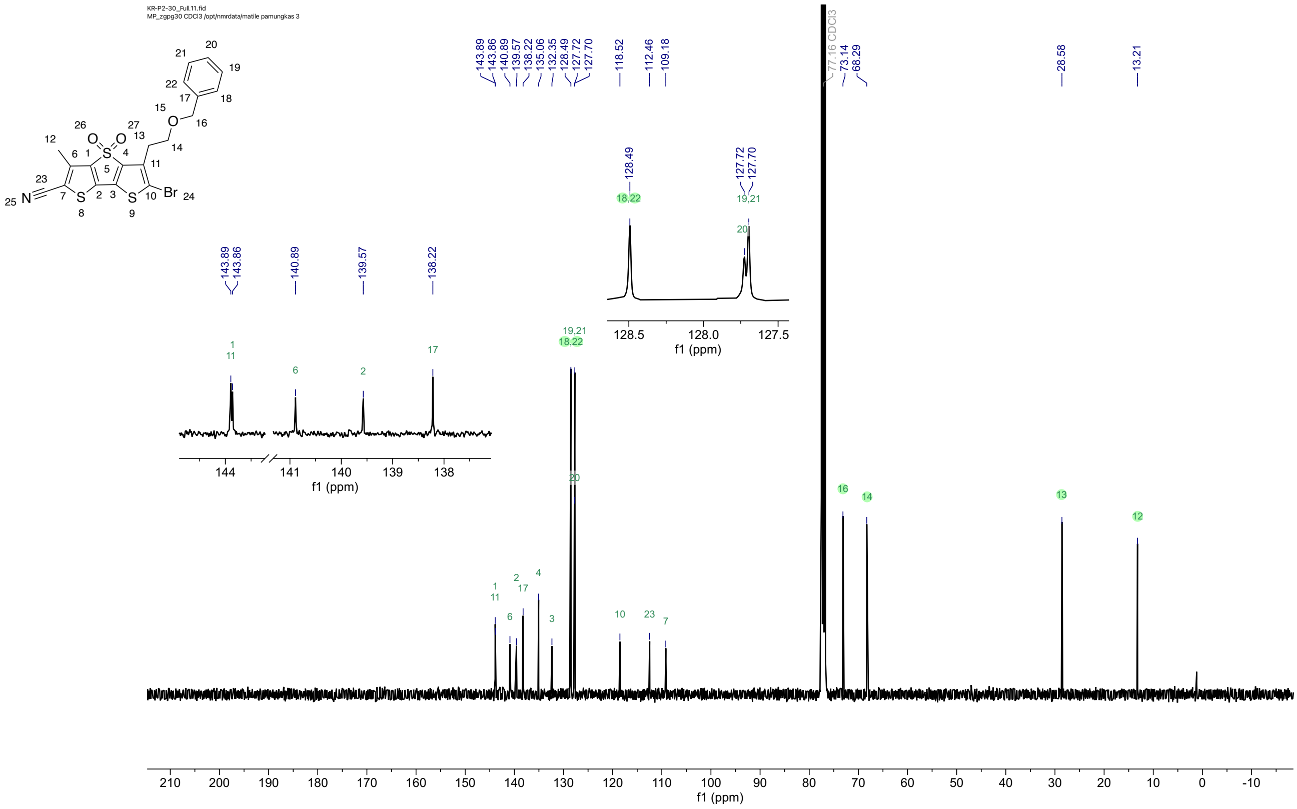

Figure S26. ^{13}C NMR (101 MHz, $CDCl_3$) spectrum of **15**.

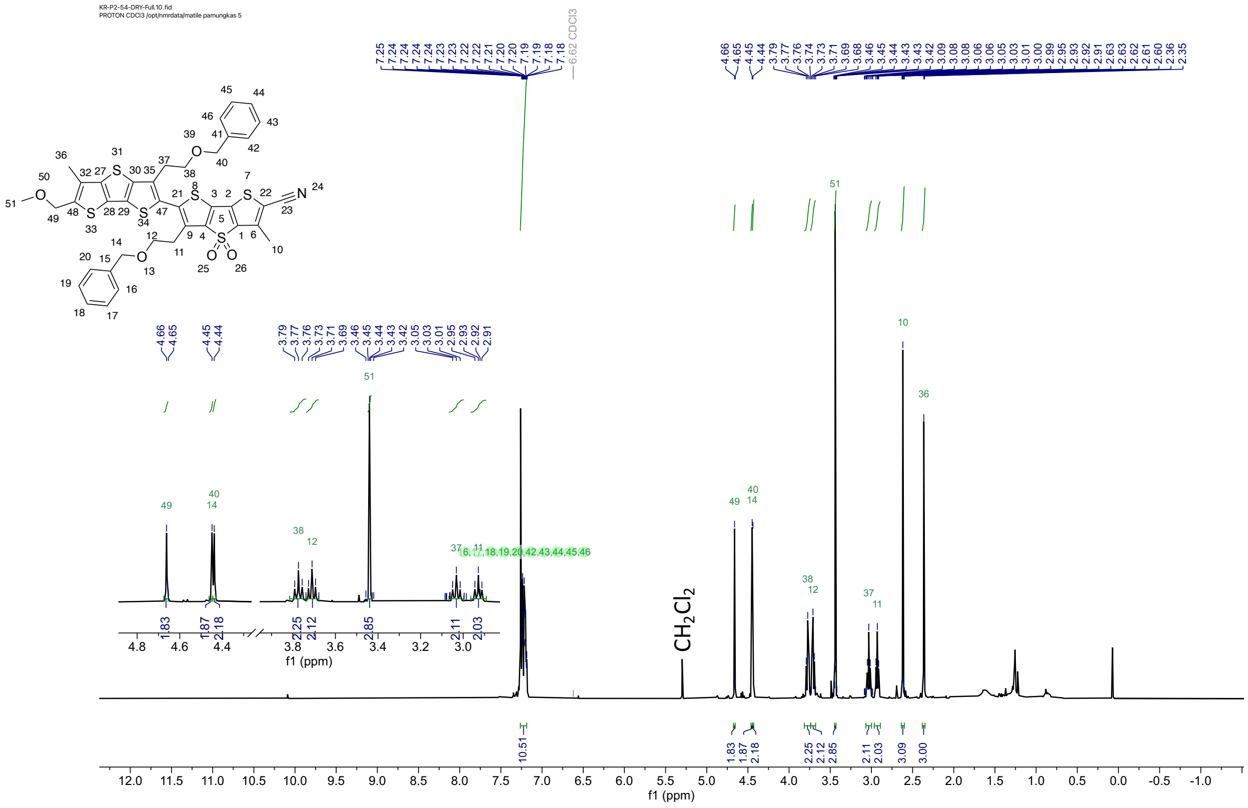

Figure S27. ^{1}H NMR (400 MHz, $CDCl_3$) spectrum of **18**.

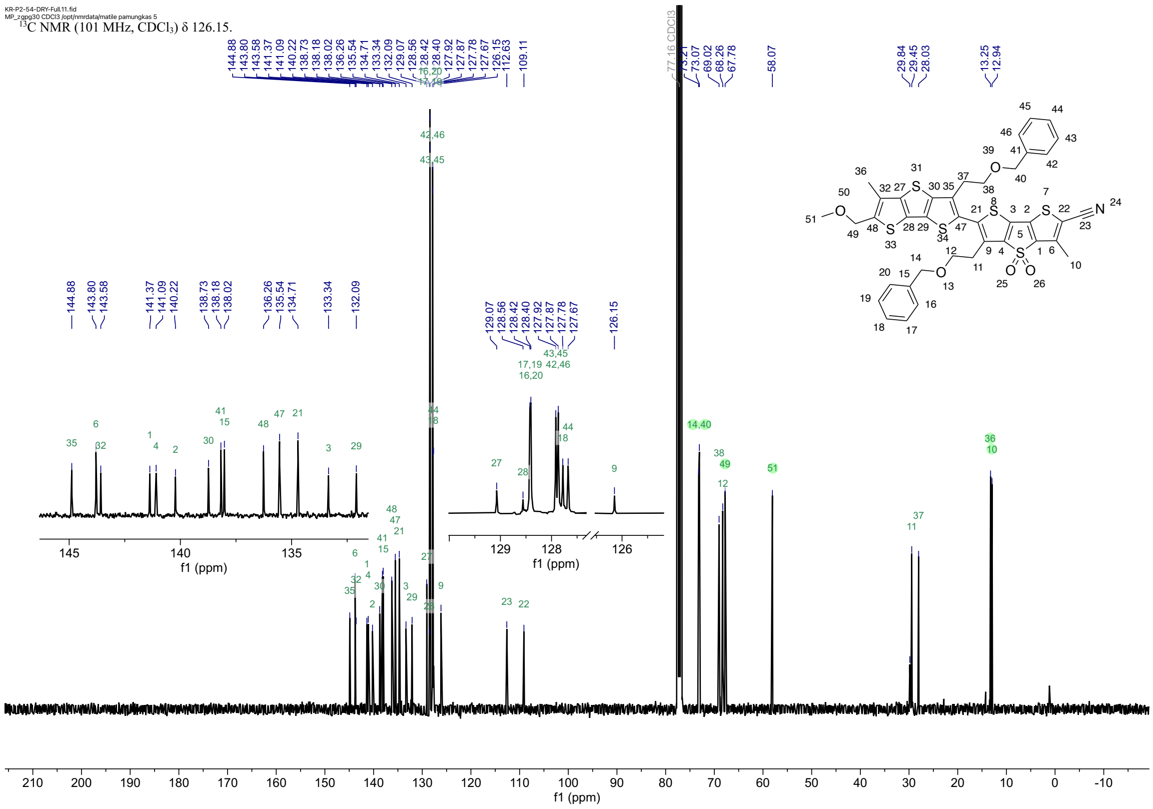

Figure S28. ^{13}C NMR (101 MHz, $CDCl_3$) spectrum of **18**.

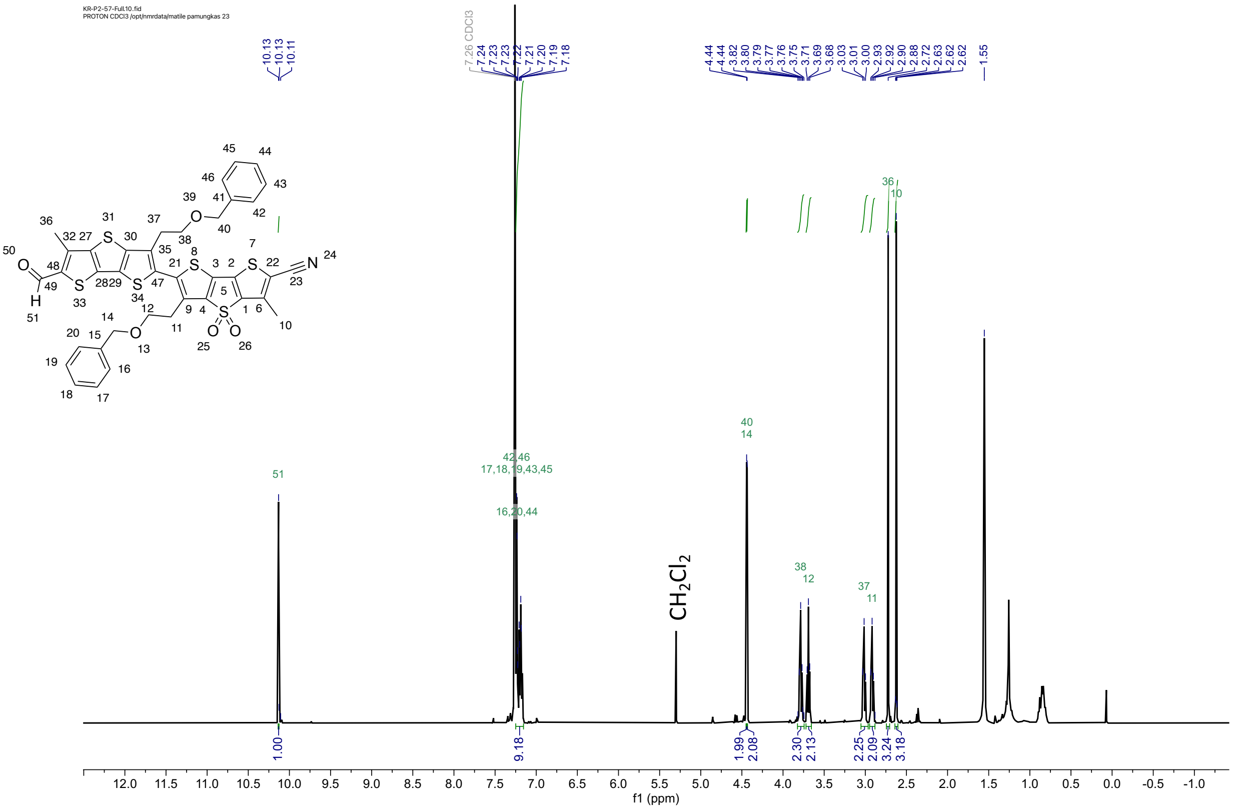

Figure S29. ^{1}H NMR (400 MHz, $CDCl_3$) spectrum of **19**.

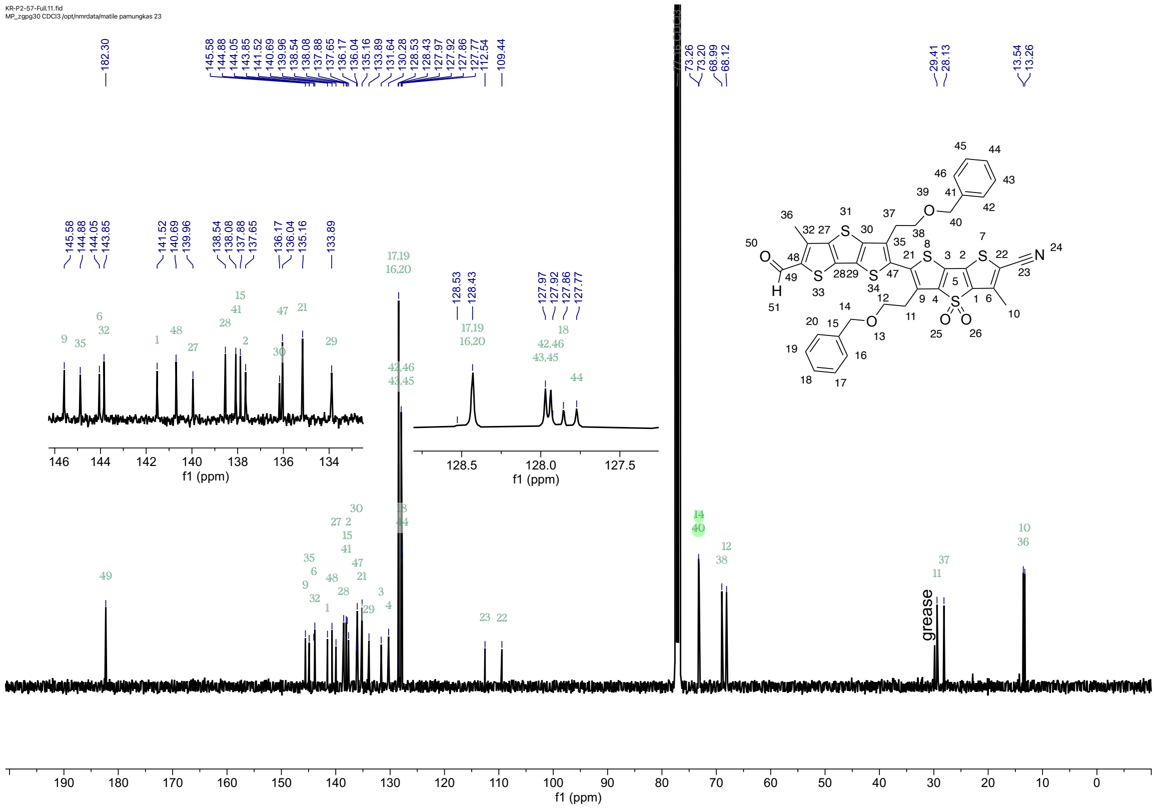

Figure S30. ^{13}C NMR (101 MHz, $CDCl_3$) spectrum of **19**.

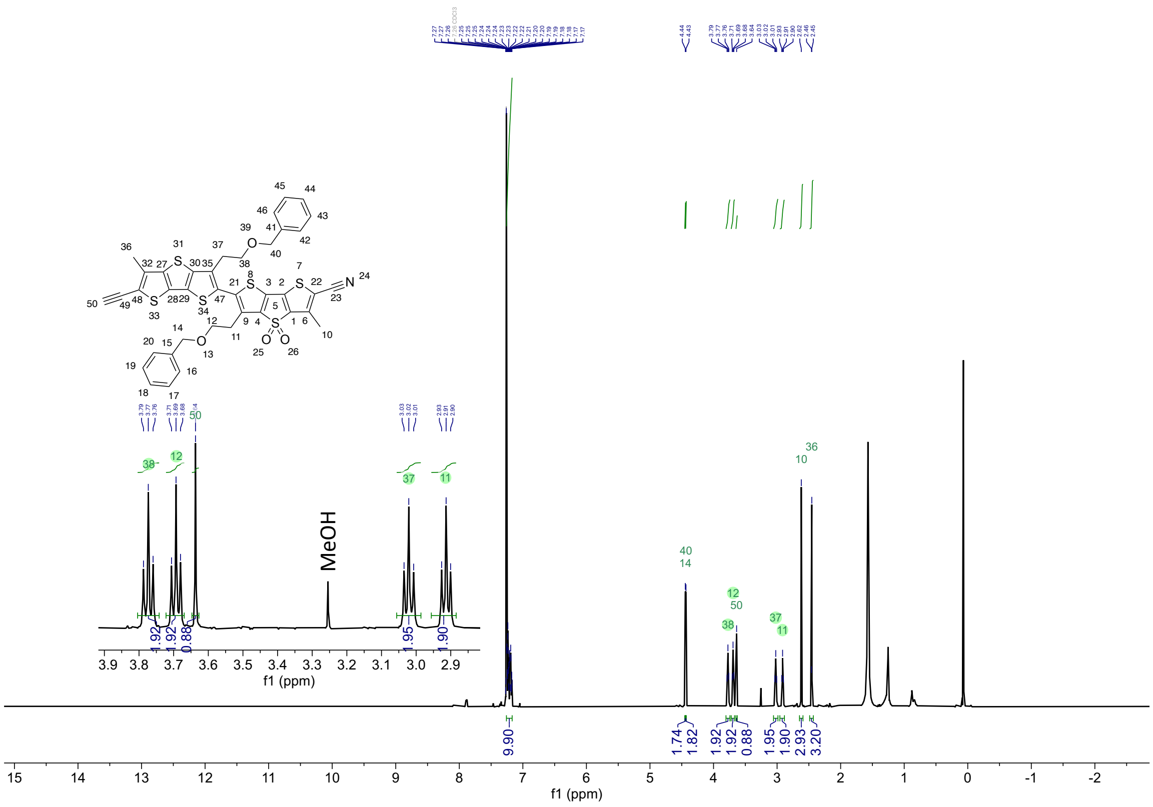

Figure S31. ^{1}H NMR (500 MHz, $CDCl_3$) spectrum of **20**.

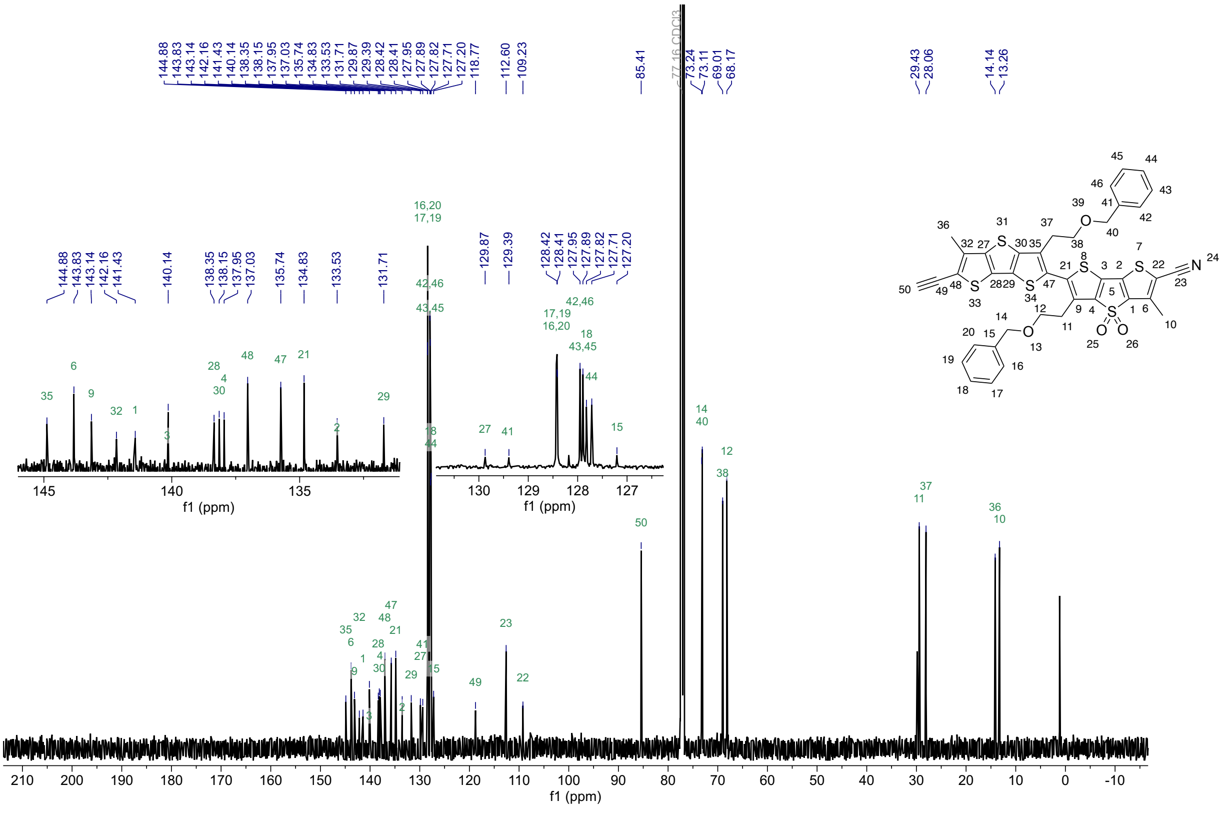

Figure S32. ^{13}C NMR (126 MHz, $CDCl_3$) spectrum of **20**.

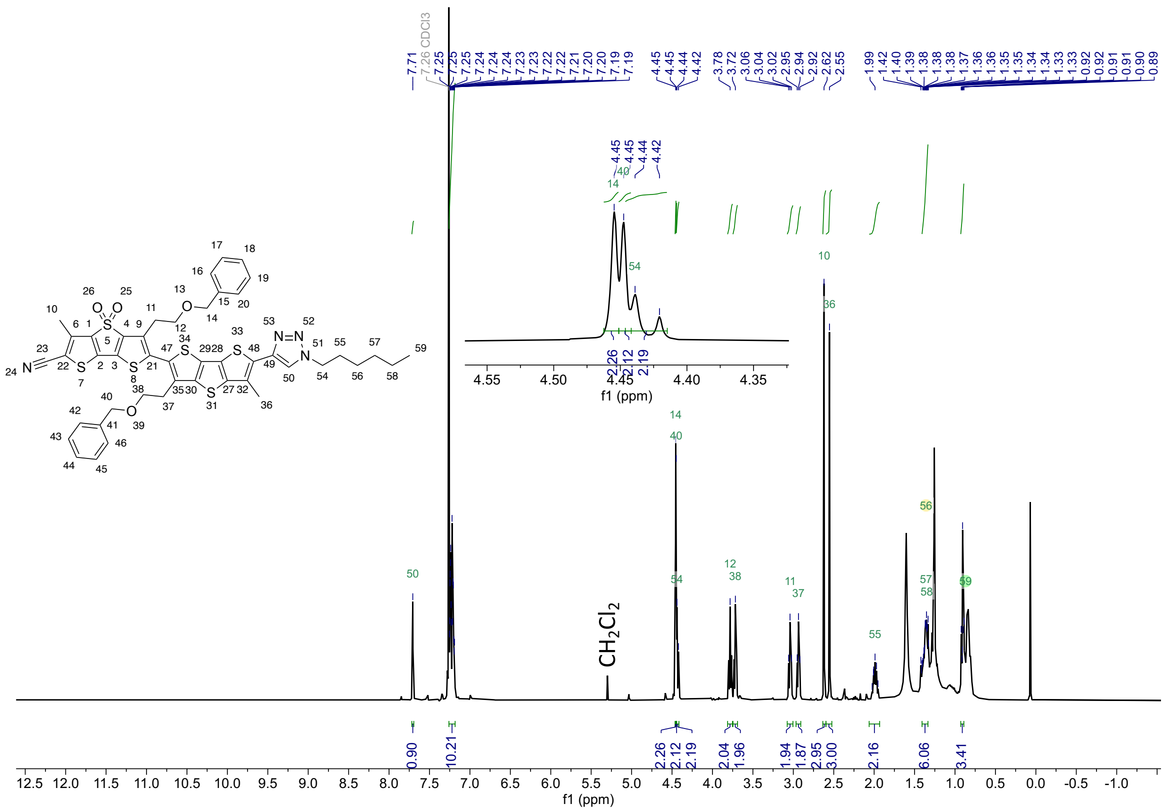

Figure S33. ^{1}H NMR (400 MHz, $CDCl_3$) spectrum of **22**.

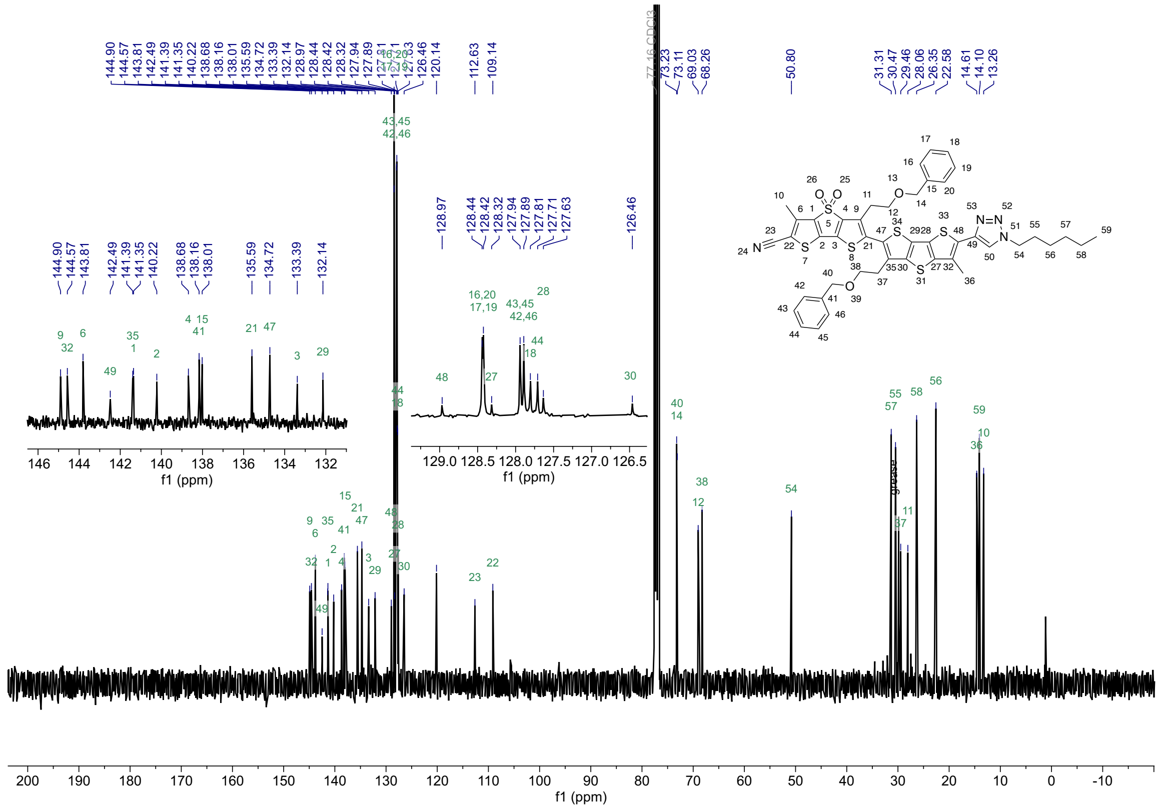

Figure S34. ^{13}C NMR (101 MHz, $CDCl_3$) spectrum of **22**.

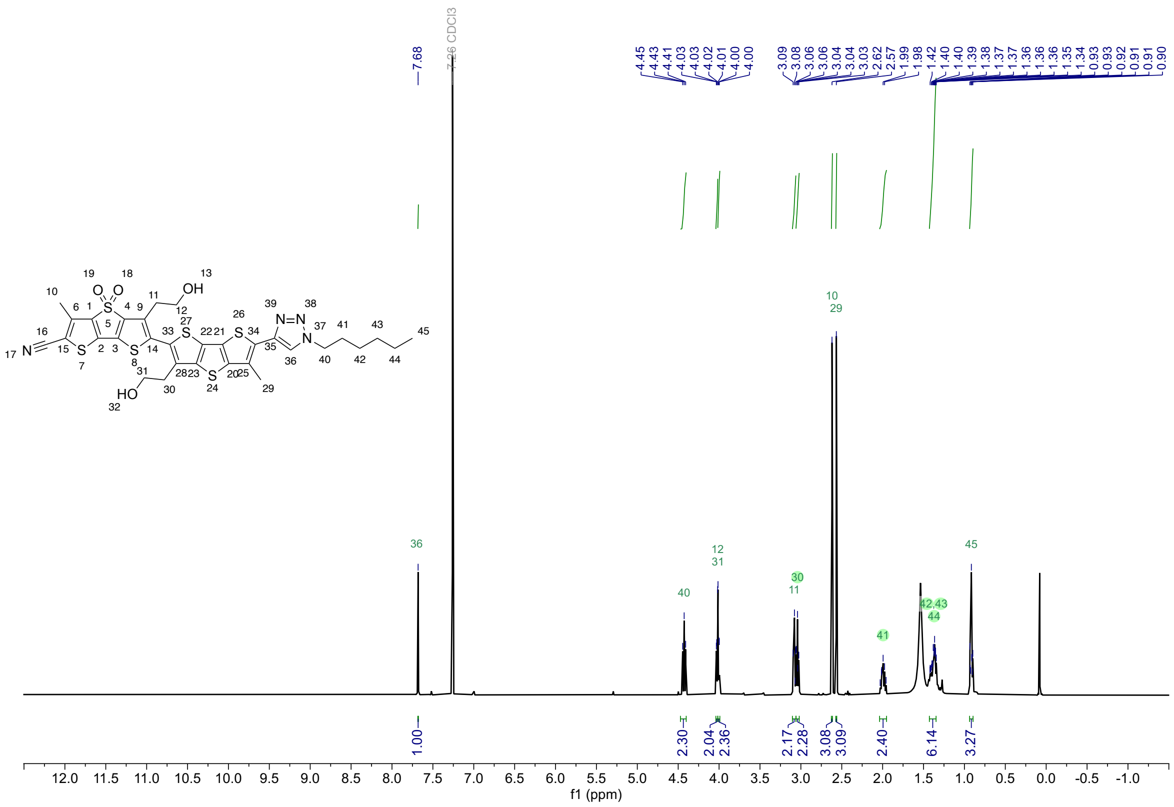

Figure S35. ^{1}H NMR (400 MHz, $CDCl_3$) spectrum of **1**.

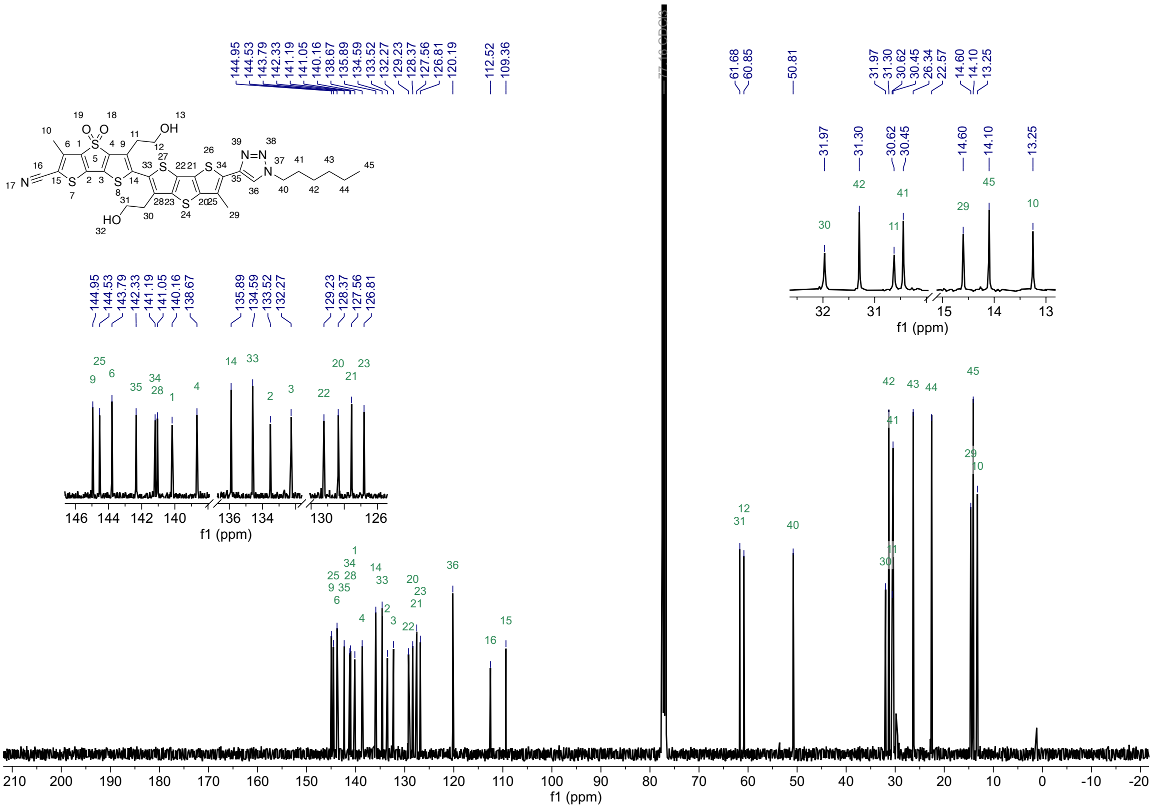

Figure S36. ^{13}C NMR (101MHz, $CDCl_3$) spectrum of **1**.

6. References

(S1) Pamungkas, K. K. P.; Fureraj, I.; Assies, L.; Sakai, N.; Mercier, V.; Chen, X.-X.; Vauthey, E.; Matile, S. Core-Alkynylated Fluorescent Flippers: Altered Ultrafast Photophysics to Track Thick Membranes. *Angew. Chem. Int. Ed.* **2024**, *63*, e202406204.

(S2) Peifer, M.; Berger, R.; Shurtleff, V. W.; Conrad, J. C.; MacMillan, D. W. C. A General and Enantioselective Approach to Pentoses: A Rapid Synthesis of PSI-6130, the Nucleoside Core of Sofosbuvir. *J. Am. Chem. Soc.* **2014**, *136*, 5900–5903.

(S3) Sinha, J.; Sahoo, R.; Kumar, A. Processable, Regioregular, and “Click”Able Monomer and Polymers Based on 3,4-Propylenedioxythiophene with Tunable Solubility. *Macromolecules* **2009**, *42*, 2015–2022.
